# Evaluation of an *E. coli* Cell Extract Prepared by Lysozyme‐Assisted Sonication via Gene Expression, Phage Assembly and Proteomics

**DOI:** 10.1002/cbic.202100257

**Published:** 2021-07-29

**Authors:** Elisabeth Falgenhauer, Sophie von Schönberg, Chen Meng, Andrea Mückl, Kilian Vogele, Quirin Emslander, Christina Ludwig, Friedrich C. Simmel

**Affiliations:** ^1^ Physics of Synthetic Biological Systems, Physics Department E14 Technical University of Munich Am Coulombwall 4a 85748 Garching Germany; ^2^ Bavarian Center for Biomolecular Mass Spectrometry (BayBioMS) Technical University of Munich Gregor-Mendel-Strasse 4 85354 Freising Germany

**Keywords:** bacterial cell extract, cell-free protein expression, mass spectrometry, phage assembly, proteomics

## Abstract

Over the past decades, starting from crude cell extracts, a variety of successful preparation protocols and optimized reaction conditions have been established for the production of cell‐free gene expression systems. One of the crucial steps during the preparation of cell extract‐based expression systems is the cell lysis procedure itself, which largely determines the quality of the active components of the extract. Here we evaluate the utility of an *E. coli* cell extract, which was prepared using a combination of lysozyme incubation and a gentle sonication step. As quality measure, we demonstrate the cell‐free expression of YFP at concentrations up to 0.6 mg/mL. In addition, we produced and assembled T7 bacteriophages up to a titer of 10^8^ PFU/mL. State‐of‐the‐art quantitative proteomics was used to compare the produced extracts with each other and with a commercial extract. The differences in protein composition were surprisingly small between lysozyme‐assisted sonication (LAS) extracts, but we observed an increase in the release of DNA‐binding proteins for increasing numbers of sonication cycles. Proteins taking part in carbohydrate metabolism, glycolysis, amino acid and nucleotide related pathways were found to be more abundant in the LAS extract, while proteins related to RNA modification and processing, DNA modification and replication, transcription regulation, initiation, termination and the TCA cycle were found enriched in the commercial extract.

## Graphical abstract

*E. coli* extracts can be prepared using a simple combination of lysozyme incubation and ultrasonication. We evaluate their performance in cell‐free protein expression via the expression of reporter proteins, the assembly of bacteriophages, and using proteomics. Our results show a synergistic effect of both lysis methods.

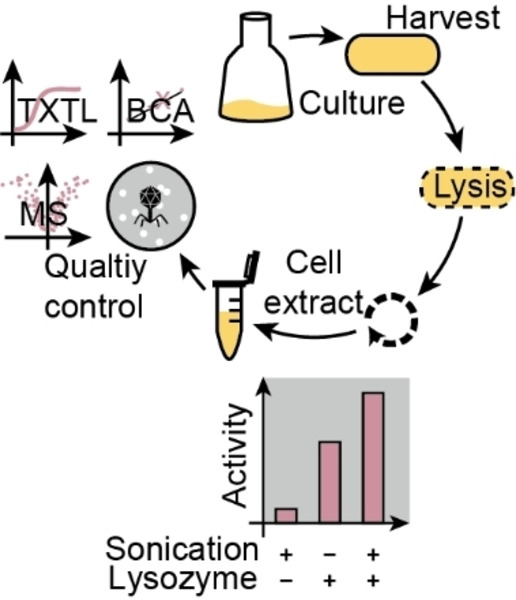

## Introduction

Cell‐free gene expression systems have become increasingly popular over the past years as tools for investigating basic biological mechanisms, for the production of proteins at high yields, rapid prototyping of components for synthetic biology, and as an integral part of synthetic cellular systems.^[1]^ Cell‐free systems are interesting as they give direct access to the biosynthetic resources of the cytoplasm and thus allow their simple utilization, manipulation, and complementation. Performing transcription and translation processes (TXTL) outside the cellular context has many advantages. For instance, they take place in the absence of the genetic background of the host and therefore the genetic machinery focuses only on the genes of interest. Furthermore, the lack of cellular growth and its associated complex gene regulatory processes and metabolism make the systems conceptually simpler to understand and quantitate.

In order to perform cell‐free gene expression reactions, the necessary biochemical machinery has to be extracted from live cells in an active form and combined with supplements (TXTL buffer) containing precursor molecules and chemical fuels. Most of the current protocols for the production of “bacterial S30 extract” – containing the soluble fraction of macromolecules including the transcription‐translation apparatus – are based on a procedure first described by Zubay.^[2]^ Various later refinements of the preparation of bacterial cell extracts or the TXTL buffer composition have achieved considerably increased protein expression yields.^[3]^ These improvements were possible through technical innovations^[4]^ and a better understanding of metabolic processes within the extract and energy regeneration systems.^[5]^ With the advent of high‐throughput methods and machine‐learning tools, further optimization of the complex reaction mixtures has become feasible.^[6]^ Cell lysis is one of the most critical steps during the production of an active cell extract. Accordingly, a wide range of lysis methods have been tested and improved for bacterial cell extract preparation in the past. Active cell extracts were produced using the bead beating technique,^[7]^ cell disruption under high pressure (using a “French press”),^[2]^ or sonication.^[8]^ Also freeze‐thaw cycles or lysozyme incubation^[9]^ were utilized, but these only resulted in extracts with a relatively low activity.

**Figure 1:**
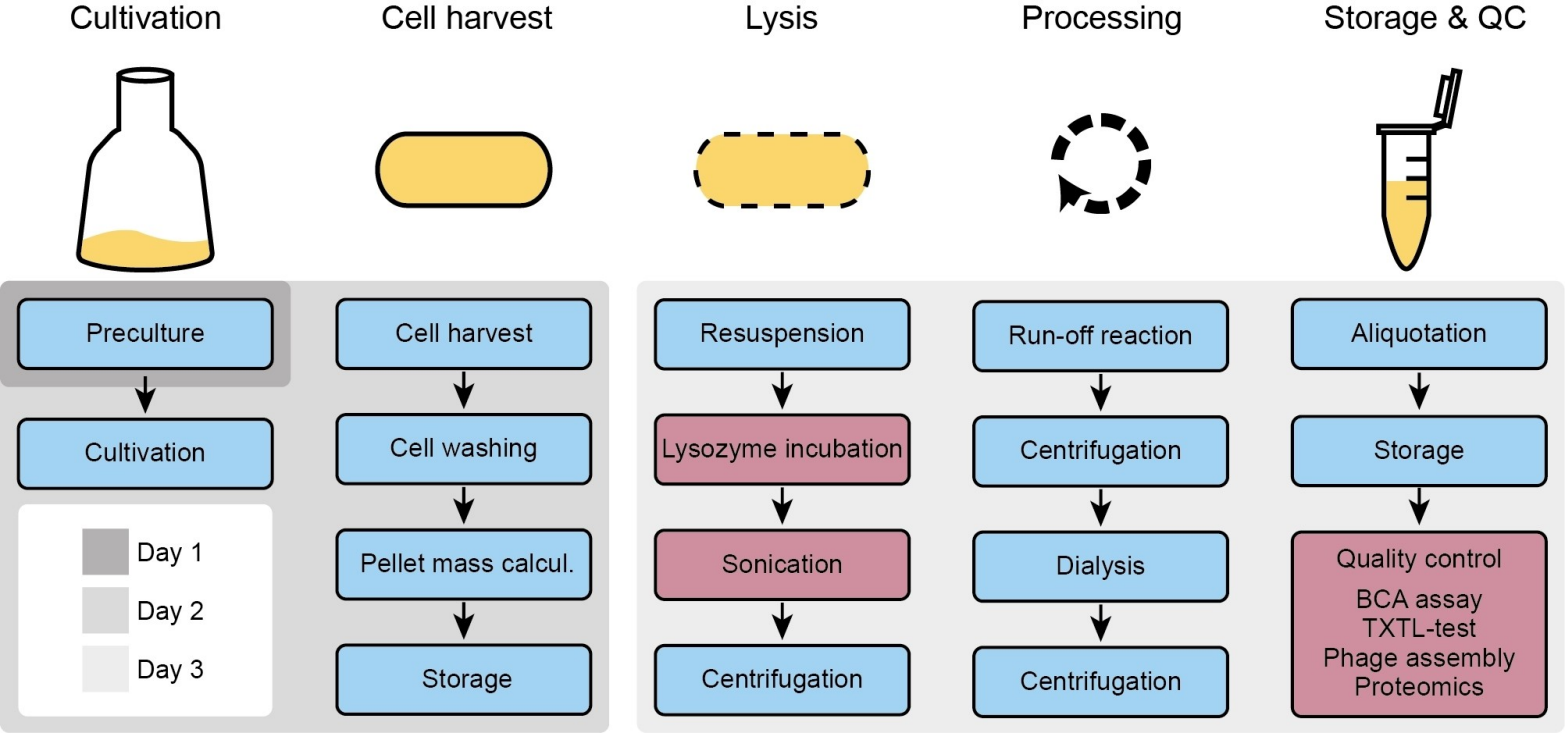
Overview of the cell extract preparation workflow, which includes cell culturing, cell harvest and washing, cell lysis, further processing steps and storage of the extract, followed by assessment of its quality. This work focused on optimizing cell lysis by using a combination of lysozyme incubation and sonication. As quality control measures, the total protein content of the produced extract was determined, the cell extract activity was measured by expressing a fluorescent reporter protein (TXTL test) and via the assembly of T7 bacteriophages. The protein compositions of selected extracts were compared to each other using quantitative proteomics.

The basic steps for cell extract preparation include cell culturing, harvesting, washing, lysing and clarifying of the cell suspensions (Figure 1). In addition, a run‐off reaction is performed, during which residual DNA and RNA is degraded and bound ribosomes are released. This is commonly followed by a buffer exchange via dialysis in order to provide optimum reaction conditions for gene expression. Preparation details vary between different labs and there are also protocols which omit some of the steps as exemplified by a high throughput protocol with just three steps.^[8]^ However, we decided to use the protocol of Sun et al.^[7]^ as a basis for our study and specifically modified its cell lysis step as it is a well‐documented protocol which details all recommended steps for cell extract preparation.

The pros and cons of existing lysis techniques can be briefly summarized as follows: Bead beating^[7]^ is a relatively inexpensive method, but time‐consuming and difficult to scale up to larger volumes. By contrast, high pressure cell disruptors allow the regulation of pressure and shear rates and thus control over the forces exerted on the bacterial cell membranes. These widely available devices are best suited for scaling up the cell suspension volumes even though the lysis comes along with a high temperature increase in the sample.^[9]^ In addition it was reported by Uhlmann et al.^[10]^ that the geometry of the seat, valve and impact ring have an influence on the cell lysis and the activity of the resulting extract, which limits the transferability of optimized protocols for one device to another device.

Also widely available laboratory instruments are ultrasound sonicators, which are commonly used for cell lysis in protein purification protocols. Kwon and Jewett^[8]^ developed a high throughput crude extract preparation method based on sonication using a 20 kHz device. They found that for optimal cell‐free protein synthesis the sonication energy input had to be adjusted depending on the volume of the cell suspension used. Small volumes were found to be very sensitive to energy input, whereas larger volumes showed a higher tolerance. In order to avoid excessive heating of the suspension, sonication is applied in bursts followed by cooling periods.

Previous attempts to produce cell lysates from *E. coli* via enzymatic cell wall degradation using 0.1 mg/mL lysozyme – without sonication – did not result in extracts with appreciable cell‐free protein synthesis,^[9]^ presumably because the peptidoglycan layer of Gram‐negative bacteria is not very accessible to the enzyme. However, Fujiwara and Doi showed that a protocol involving lysozyme incubation combined with osmotic shock and freeze‐thaw cycles does result in an active cell extract.^[11]^ We therefore reasoned that lysozyme treatment might be useful to weaken the bacterial cell wall just enough to allow ultrasonic cell disruption at lower energy inputs than lysozyme‐free sonication protocols.

In the present work, we show that lysozyme treatment with higher lysozyme concentrations and modified reaction conditions compared to Shrestha et al.^[9]^ can be used to support a gentle sonication protocol. In particular we avoided an additional incubation step of the cell suspension at a physiologically relevant temperature (which can have a counterproductive result) by performing the lysozyme incubation on ice instead at 37 °C. As a result, the gene expression capability of our extract was considerably improved and comparable to a commercially available extract. In our experiments, we found that fluorescent proteins can be expressed from a constitutive promoter up to a concentration of 22 μM, which corresponds to 0.6 mg/mL. Using a different promoter with higher promoter strength can further increase the gene expression yield. As another example, we produced and assembled functional T7 bacteriophages in our extract, which resulted in higher phage titers than with any other *in vitro* transcription/translation system we tested. In order to rationalize our observations, we analyzed selected extracts using state‐of‐the‐art quantitative proteomics and focused on differences in protein composition resulting from different culture conditions and different lysis methods. Our analysis indicates a better release of DNA binding enzymes with increasing numbers of sonication cycles. Major differences were detected compared to a commercial extract, which is of particular interest as the extracts show a similar performance in cell‐free gene expression experiments.

## Results and Discussion

### Preparation of cell extract via lysozyme‐assisted sonication (LAS)

An overview of our general workflow for lysozyme/sonication‐based preparation of cell extract is shown in Figure 1. The most important deviation from other protocols is the lysis step, in which freshly resuspended cells (after cell harvest) are treated with varying concentrations of lysozyme and then subjected to multiple sonication cycles. A detailed protocol is given in the methods section.

### Cell‐free expression of fluorescent proteins and protein content of cell extracts

In an initial study with shaking flask cultivation (“SF batches”), we harvested the cells in the exponential growth phase (late log phase) at an OD between 1.8 and 2 (Figure S3D&E). We coarsely screened the influence of lysozyme incubation against the number of sonication cycles. In the following, we use a shorthand notation for the lysis conditions used, where Sx/Ly denotes a protocol with x sonication cycles at a lysozyme concentration of y mg/mL. For all extracts, the total protein content was determined by a Bicinchoninic acid (BCA) assay, while the efficacy of cell‐free gene expression was tested by expressing the fluorescent reporter protein YFP (mVenus) from a constitutive promoter J23106.

We also tested other reporter proteins within the same expression cassette namely a RFP reporter (mScarlet‐I), a GFP reporter (GFPmut3) and a CFP reporter (mTurqoise2). We observed appreciable differences in expression levels and yields in a range spanning one order of magnitude (Figure S6). These may in part be explained by the optimized codon usage for the YFP reporter and differences in maturation path and time, quantum yield and other protein characteristics. Secondary structure and GC content of the corresponding mRNA can also have an influence on protein expression.^[12]^ Nevertheless, the different reporters showed the same trends in the screening experiments for the different lysis settings. In addition, the maximum protein expression rate and the end level of expressed protein concentration were positively correlated (Figure S5). We therefore restrict the following discussion of gene expression efficiency to the concentration end level of the YFP reporter.

**Figure 2:**
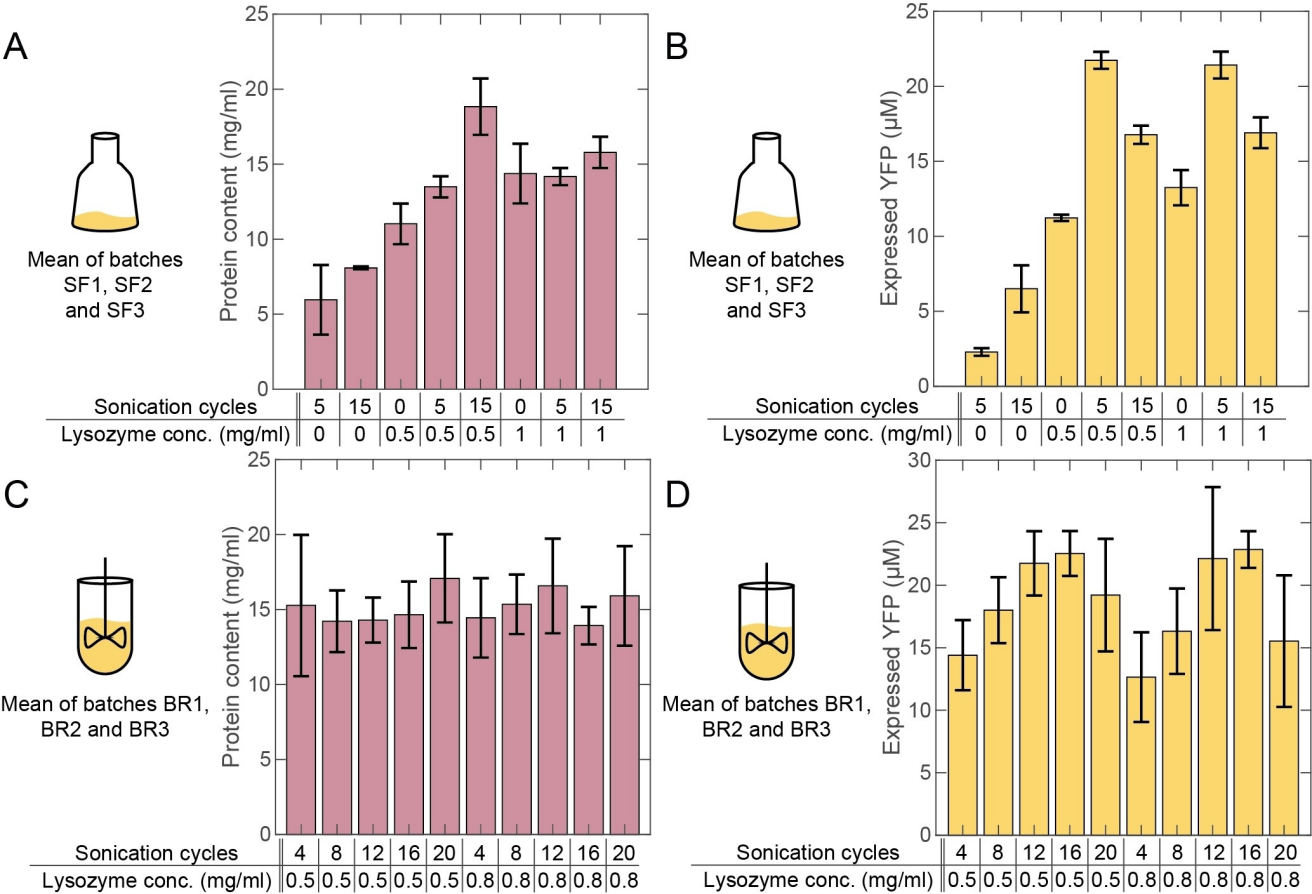
(A) The mean total protein content was determined for three biological shaking flask replicates, which were lysed using a lysozyme concentration of 0, 0.5 or 1 mg/mL in combination with 0, 5 or 15 sonication cycles. (B) TXTL‐test for the shaking flask replicates. The mean end levels are presented for a YFP expressed in the shaking flask cell extracts. (C) Mean total protein content for three bioreactor replicates, which were lysed using 0.5 or 0.8 mg/mL lysozyme in combination with 4, 8, 12, 16, or 20 sonication cycles. (D) TXTL‐test for the bioreactor replicates. The mean end levels are shown for a YFP reporter expressed in the bioreactor cell extracts.

Samples, which were not incubated with lysozyme but lysed with sonication pulses (S5/L0) had the lowest mean protein content of around 6 mg/mL measured in the BCA assay (Figure 2A). These samples also generated the lowest fluorescence signal intensities in TXTL experiments (Figure 2B). The increase in the number of sonication cycles (from 5 to 15) resulted in an increase in the mean protein content (mean of 3 biological replicates) and also in an increase of the fluorescence end level almost by a factor of 3. Compared to these samples we observed a higher protein content and therefore better lysis efficiency and also a higher TXTL fluorescence end level for the S0/L0.5 and S0/L1 samples, which were not sonicated at all but incubated with lysozyme. Both tested lysozyme concentrations (0.5 mg/mL and 1 mg/mL) resulted in similar signal intensities.

As stated above, we had initially surmised that a combination of lysozyme incubation and sonication should have a synergistic effect, as lysozyme is supposed to weaken the integrity of the cell walls and thus support sonication‐induced cell lysis. Surprisingly, combined protocols did *not* result in an increased lysis efficiency (the mean cell extract protein content of three biological replicates saturated between 14 and 19 mg/mL, Figure 2A), but nevertheless in a twofold YFP increase in the TXTL test, when comparing the S5/L0.5 to the S0/L0.5 sample. We observed the same end level for both tested lysozyme concentrations (compare S5/L0.5 and S5/L1, Figure 2B). The fluorescence end level was reduced for the 15 cycle samples, suggesting an optimum intermediate number of sonication cycles, which was also confirmed using a t‐test (Table S4).

We next used cells produced from a 2 L fed‐batch culture in a bioreactor (“BR batches”), which were again harvested in the exponential growth phase. By this we increased our pellet mass yield by a factor of 4 and we were able to expand our screening range. We tested lysozyme concentrations of 0.5 mg/mL and 0.8 mg/mL against 4, 8, 12, 16 and 20 sonication cycles (Figure 2C and 2D). The BCA assay revealed that all cell extracts had a similar mean protein content around 15 mg/mL. In contrast to the shaking flask extracts, the deviations between the biological replicates were higher than the deviations between the different lysis settings within a single replicate (Figure S4). We again performed TXTL tests and compared the corresponding fluorescence levels. For both lysozyme concentrations an increase in the number of sonication cycles resulted in an increase in the YFP end level where an optimum was reached in the range of 12–16 cycles with end level concentrations again up to 22 μM. The optimum was again proven to be significant using a t‐test (Table S4).

### Batch‐to‐batch variations

Despite the many advantages of cell‐free protein expression studies, they potentially suffer from considerable batch‐to‐batch variations, which depend on details of the cell extract preparation procedure. Variability can result from variations in culture conditions, the growth state of the cells during harvest, cell suspension viscosity, energy input or heat production during lysis, lysis efficiency and activity of the extracted proteins.^[13]^ In our shaking flask extracts we observed standard deviations in the range of 2 % and 24 % of the signal intensity for three biological replicates, with a mean at 8 %. For the bioreactor‐prepared extracts we observed slightly higher deviations between the biological replicates in the range of 6 % to 34 %, but the mean was still at 19 %. This might be a result of the differences in growth curves monitored for the bioreactor batches (extended lag phase for two of the three batches, cf. Figure S3). In our hands, common procedures such as careful monitoring of cell growth and harvesting point of the cells, cooling of the cell suspension over the full preparation time and mixing during cell lysis turned out to be sufficient for acceptably small batch‐to‐batch variations.

### Comparison to other extracts and preparation methods

**Figure 3:**
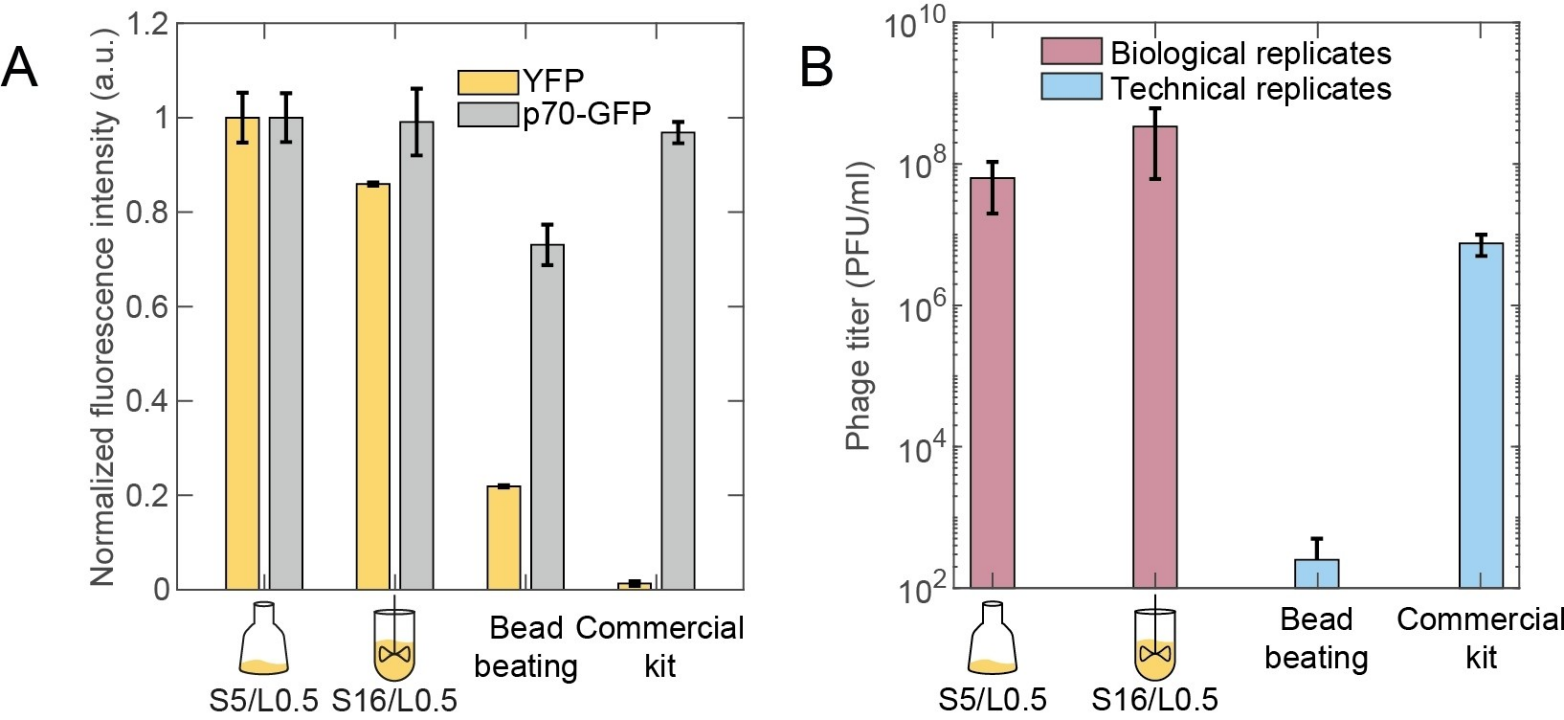
(A) The shaking flask replicates (S5/L0.5) and bioreactor replicates (S16/L0.5) were tested in comparison to a bead beating batch and a commercial kit. In addition to our self‐prepared YFP plasmid we tested the p70‐GFP control plasmid, which codes for GFP under the control of a lambda promoter. The fluorescence signals were normalized to the maximum measured signal for each plasmid. (B) Plaque assays for the best shaking flask and bioreactor replicates, a commercial extract and a beat beating batch.

Our best shaking flask replicates (S5/L0.5) and bioreactor replicates (S16/L0.5) were tested in comparison with a self‐prepared batch using a bead beating protocol^[7]^ and a commercially available kit (myTXTL Sigma 70 Master Mix Kit, Arbor Biosciences) (see Figure 3A). In addition to our YFP plasmid we tested the pTXTL‐p70a(2)‐deGFP HP control plasmid shipped with the commercial kit, which codes for a GFP expressed from a lambda promoter. Our standard mVenus reporter has a higher quantum yield, brightness and codon adaptation index than the GFP from the commercial control plasmid. On the other hand, the computationally predicted translation rate^[14]^ for the commercial GFP is higher by a factor of 10, which is mainly ascribed to the stronger secondary structure at the 5′UTR of our reporter mRNA transcripts (or standby sites,^[14]^ see Table S3 for a detailed comparison). The fluorescence signals were normalized to the maximum signal measured for each plasmid (see Figure S7B for raw data). In the bead beating batch, we only observed 22 % of the maximum signal for our YFP plasmid and 71 % of the maximum for the p70‐GFP plasmid.

Furthermore, in the commercial kit we obtained a mere 1 % of the maximum signal for our self‐prepared YFP plasmid, while a 94 % signal was measured for the kit's control plasmid. We surmised that residues from our plasmid preparation (following a standard phenol‐chloroform extraction protocol) might have a detrimental effect on the commercial cell extract, as we observed also low signals for our RFP, GFP and CFP reporter plasmids in the commercial extract (Figure S7A). We thus purified the p70‐GFP control plasmid using a simple phenol‐chloroform extraction protocol and repeated the TXTL test in the commercial kit, which indeed resulted in lower signal levels (Figure S7C).

### Comparison to the protocol by Kwon and Jewett^[8]^ and the protocol by Fujiwara and Doi^[11]^

Whereas in the study of Kwon and Jewett the required sonication energy input depended just on the cell suspension volume, we observed different optima for different culture methods, e. g., S5 L0.5 for shaking flask and S16 L0.5 for bioreactor cultivation. Further, we only used 404 J energy input for 5 cycles and 1.3 kJ for 16 sonication cycles compared to the 2.2 kJ expected from their study for a 4 mL cell suspension volume, but we supported the lysis step using lysozyme. As a result, our total extracted protein content was smaller (about 15 mg/mL compared to 40 mg/mL). Potentially, a fraction of non‐lysed *E. coli* might be present after the lysis step in our protocol, but these cells would be removed during the centrifugation steps (in total 3) in the following extract processing steps. In addition, lysozyme is neither deactivated nor removed from the extract which makes survival of any remaining *E. coli* appear unlikely. On the other hand, the lower energy input used in our protocol potentially benefits the activity of the enzymes contained in the extract. In fact, the gene expression capability of our extract was similar as for the extract by Kwon and Jewett, as we reached a maximum YFP end level of 0.6 mg/mL expressed from a constitutive *E.coli* promoter at a plasmid concentration of 3 nM (5.7 μg/mL) (compared to 1 mg/mL for expression from a much stronger T7 promoter at a plasmid concentration of 13.3 μg/mL^[8]^).

In contrast, Fujiwara and Doi presented a protocol based on a combination of osmotic shock, lysozyme incubation and freeze thaw cycles. They reached a cell extract protein content in the range of 20–30 mg/mL and a protein expression yield of 10–20 μM (0.25–0.5 mg/mL) using either a template concentration of 1.5 nM with a T7 promoter or 10 nM with a OR2OR1 promoter. A more detailed comparison to both studies and also to the study by Sun et al. can be found in Table S5.

### Assembly of bacteriophages in the cell‐free system

As an alternative to the synthesis of fluorescent proteins, we also assessed the quality of our cell extract via *in vitro* expression and assembly of T7 bacteriophages.^[4b]^ Phage assembly is a considerably more complex process than the expression of just a single protein[^4b^, ^15^] and can thus serve as a benchmark for the capability of the cell extract to support more complex biochemical processes. For quantitation of phage assembly, we performed plaque assays and determined the phage titers. In general, the infection titer depends on the concentration of phage particles, the ratio of phage particles compared to host cells, the physiological state of the host cell (competence) and the activation state (stress versus hunger/ feast state). These parameters are difficult to control over several experiments, and we therefore performed a single experiment to compare the best lysis setting of our shaking flask (S5/L0.5) and bioreactor replicates (S16/L0.5) with the phage titers reached for the self‐made bead beating batch and the commercial expression kit. All LAS extracts showed high phage titers up to 10^9^ PFU/mL (with a mean of 10^8^ PFU/mL for three biological replicates; Figure 3B and Figure S8). This has to be compared to a mean titer of close to 10^7^ PFU/mL for the commercial kit and merely 250 PFU/mL for the bead beating batch.

Overall, our results show that there is an optimum number of sonication cycles for both cultivation methods (5 for shaking flask and 12–16 for bioreactor samples). Bacteria grown in the bioreactor – where they are subjected to larger shear forces than in shaking flask culture – have been previously found to change their morphology (resulting, e. g., in an increase in cell length^[16]^), which potentially allows them to sustain a larger number of sonication cycles. When expressing fluorescent proteins in extract prepared by the LAS protocol, a higher YFP end level was reached compared to a bead beating cell extract, but similar end levels were reached as in a commercial extract and in the study of Kwon and Jewett. Notably, our cell extract had significantly higher gene expression yields for plasmids which were purified with an inexpensive phenol chloroform precipitation (compared to the commercial kit and the bead beating batch). In the plaque assays our LAS extracts performed marginally better than the commercial extract and the bead beating batch again showed the worst results.

### Proteomics

In order to elucidate the molecular basis for the observed differences in performance, we analyzed selected cell extract samples and technical replicates of the commercial extract using state‐of‐the‐art quantitative proteomics similar to the study of Garenne et al.^[17]^ We chose the best performing shaking flask extracts (biological replicates SF1, SF2, SF3 with lysis setting S5/L0.5) and prepared and analyzed bioreactor samples with the same lysis settings (three biological replicates with setting S5/L0.5) to identify differences in protein composition that are correlated with the different culture conditions. We also analyzed shaking flask samples S0/L1, S5/L1 and S15/L1 to find differences in protein composition resulting from different lysis settings.

**Figure 4:**
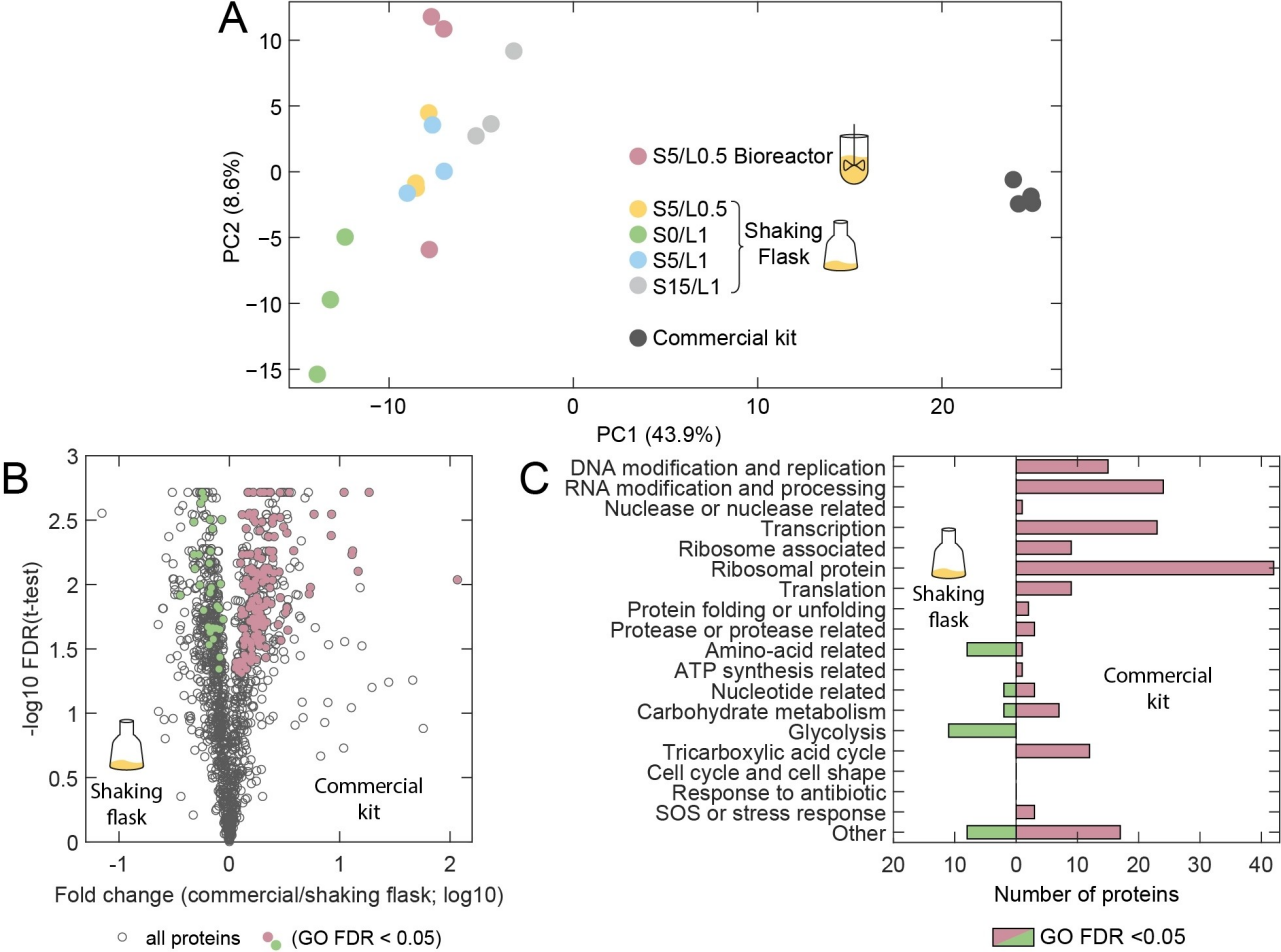
Comparison of the proteomes of extracts prepared from bioreactor and shaking flask cultures and a commercial kit. (A) The principal component analysis (PCA) shows that the proteome of the commercial extract is distinct from those of self‐made extracts (separated on PC1, accounting for 44 % of the total variance). (B) Volcano plot of shaking flask samples S5/L0.5 against the commercial kit. Proteins with a FDR below 0.05 and proteins exclusively found only in shaking flask preparations or in commercial extract were subjected to an enrichment analysis. Proteins which were assigned to GO terms with an FDR below 0.05 are highlighted in the plot. (C) Enrichment analysis derived from the comparison shown in (B). The significant proteins were assigned to keywords related to gene expression and energy regeneration.

We quantified around 1500 proteins in each preparation. A principal component analysis (PCA) showed a systematic difference between the proteomes of self‐made extracts (biological replicates) and the commercial kit (technical replicates), which were separated on principal component 1 (PC1), explaining 44 % of the total variance (Figure 4A). Self‐made extracts scattered on PC2 (explaining 8.6 % of the total variance). Compared to extracts prepared with sonication, the S0/L1 extract resulted in more negative PC1 values, indicating an influence of sonication on the proteome content of the extracts. This is in agreement with the results of the TXTL tests, as the samples prepared without sonication also had a reduced expression yield. Samples S5/L0.5 and S5/L1 overlapped in the PCA plot, which demonstrates a similar proteome composition and is consistent with the comparable TXTL test results. Motivated by these results, we decided to further investigate the proteomic differences between different extracts using t‐tests and further analyzed proteins with a false discovery rate (FDR) in the t‐test below 0.05 and ‘unique proteins’. Unique proteins are present in all three replicates of one extract but absent in all of the three replicates of the other extract (these proteins cannot be represented in a volcano plot). The distinctive proteins were then subjected to a gene ontology enrichment (GO) analysis to identify whether specific GO terms were statistically enriched. Proteins found in GO terms with an FDR<0.05 were subsequently roughly classified to keywords using the UniProt database^[18]^ to simplify the representation.

In the first step we analyzed the differences between the extracts produced from shaking flask and bioreactor cultivation (Figure S9). The protein content of the two preparations was largely similar, except for three unique proteins in the shaking flask, which were found in GO terms with GO FDR<0.05 that could be assigned to anaerobic growth conditions. This is a somewhat expected result as in contrast to bioreactor cultivation we did not provide additional oxygen in shaking flask cultivation.

We next compared batches, which were lysed with different sonication cycles at the same lysozyme concentration of 1 mg/mL (S0/L1 vs. S5/L1 and S0/L1 vs. S15/L1, Figure S10A and S10B). In total we subjected 2, 27, 4 and 53 proteins to GO enrichment analysis and found just 7 (S5/L1) and 21 (S15/L1) proteins in significant GO terms (see Table S10–12 for protein names and Table S13 for the number of analyzed proteins in each comparison). For both comparisons these could be assigned to keywords related to DNA replication, relaxation, repair or recombination or were transcriptional regulators. For the gene expression yield this may play just a minor role, but it indicates that increased sonication energy input results not just in increased lysis efficiency but also in a more efficient release of proteins bound to the genome, which would be otherwise lost together with the genome during the first centrifugation step.

In the next step we compared our extracts against the commercial kit (Figure S10C–F and Figure 4B and C). Here one has to keep in mind that we have no detailed information about the preparation of the commercial extract, so that any observed deviation could be the result of differences during each single step of the preparation workflow. However, as the YFP expression capability of our extracts and the commercial system are very similar, it is still of interest to compare the differences between the proteomes of the extracts.

As our analysis of the bioreactor replicates S5/L0.5 and the shaking flask replicates S5/L0.5 revealed only minor differences, one would expect similar results for the comparison of either BR or SF samples against the commercial extract, respectively. In fact, however, the results are found to be quite different, because the bioreactor replicates show a higher variance among each other (as noted above), which results in higher p‐values and thus an exclusion of the corresponding proteins in the enrichment analysis. As a consequence, the commercial and bioreactor extracts differ in abundance of just 8 and 5 proteins with FDR<0.05 and showed 30 and 121 unique proteins, respectively. After enrichment analysis we further assigned 11 proteins to keywords, which were related to amino‐acid biosynthesis, a nuclease or not relevant for cell‐free gene expression (see Figure S10C and D).

In contrast, the commercial and shaking flask extracts differed in the abundance of 309 versus 356 proteins with FDR<0.05 (corresponding to −log_10_(FDR)>1.3) and had 116 and 53 unique proteins, respectively (see Figure 4B and C). After enrichment analysis in total 172 and 31 proteins were further analyzed and assigned to keywords, respectively (40 % and 8 % of the proteins which had an FDR<0.05 or were unique proteins). According to this analysis, in shaking flask batches proteins taking part in carbohydrate metabolism and glycolysis, amino‐acid and nucleotide related pathways were found to be more abundant than in the commercial kit. In contrast, proteins which are related to RNA modification and processing, DNA modification and replication, transcription regulation, initiation and termination and the TCA cycle were found enriched in the commercial extract. The potential role of the single proteins found in these keywords is discussed in the Supplementary Information in detail. In short one can summarize, that ATP regeneration might be upregulated in our extract, if intermediates of the glycolysis pathway are used as an energy source. In addition, one can potentially use inexpensive precursors to produce amino acids inside the extract by taking advantage of the enriched proteins of the amino acid biosynthesis pathways. On the other hand, lysis efficiency (higher degree of fragmentation and release of DNA bound proteins) potentially is higher for the commercial extract, as some transmembrane proteins and DNA binding proteins were found to be enriched in this extract. In addition, also the translational capacity appeared to be enhanced in the commercial system as we found a higher abundance of ribosomal proteins and elongation factors. However also some stress factors, ribonucleases and proteases were found to be enriched in the commercial extract, which might have a negative effect on gene expression yield.

In general, the results of this analysis have to be interpreted with care. Notably, 67 % of the compared proteins only had a fold change of less than 2 (see Figure S10F). In combination with the missing information about the activity of the single enzymes, the effect on gene expression yield is difficult to assess.

## Conclusion

In conclusion, we have studied a cell extract preparation protocol, which uses a combination of lysozyme incubation and multiple sonication cycles. In contrast to earlier work, we observed a synergistic effect of lysozyme incubation and sonication on the expression efficiency of the cell extract. Expression of YFP from a self‐prepared reporter plasmid in our cell extract resulted in a 100‐fold higher fluorescence signal than when using a commercial CFS. Using a commercial control plasmid, by contrast, resulted in similar signals in both types of extract, suggesting a sensitivity of the commercial product towards residues from the plasmid purification protocol used. Our lysozyme incubation/sonication‐based extract performed better in the *in vitro* assembly of T7 bacteriophages, which we used as an example of a more complex assembly process. Our cell extract reached a phage titer, which was at least one order of magnitude higher than what could be obtained in a commercial system. By contrast, phage assembly using a bead beating protocol (instead of sonication) resulted in a very low titer.

We attempted to rationalize the observed differences using state‐of‐the‐art quantitative proteomics. This approach revealed rather small differences among our self‐made batches. We found that higher sonication energy inputs might not just increase the lysis efficiency but potentially also promotes the dissociation of DNA‐binding enzymes from DNA. In addition, we compared our extracts to a commercial system. Even though both our self‐made and the commercial cell extract were prepared from *E. coli* Rosetta 2 (DE3) cells^[17]^ we expected to observe differences in the abundance of proteins due to the different culture and lysis methods. Indeed, our proteomic analysis showed clear differences between the commercial cell extract and our batches. However, lack of information about the *activity* of the enzymes (rather than their abundance) limits the interpretability of these results.

A range of other factors might come into play that influence expression yield. For instance, it has been reported that *in vitro* protein expression is limited by low ribosomal activities and in particular by the lack of ternary complexes formed by EF−Tu, tRNA and GTP.^[19]^ Further, energy metabolism has been shown to play a crucial role in protein synthesis yield.[^3a^, ^20^]

## Experimental Section

A detailed experimental section is given in the Supporting Information.

## Proteomics data availability

The mass spectrometry proteomics data have been deposited to the ProteomeXchange Consortium (http://proteomecentral.proteomexchange.org) via the PRIDE^[21]^ partner repository with the dataset identifier PXD024458.

## Contributions

E.F., S.v.S. and F.C.S. planned the project. E.F. and S.v.S. prepared the cell extract batches and performed and analyzed the TXTL tests and BCA assays. K.V. prepared the phage assemblies. C.L. and Q.E. performed the MS measurements. C.M., E.F. and A.M. analyzed and interpreted the MS data. E.F., A.M., C.M., F.C.S. and C.L. wrote the manuscript and all authors discussed the results and commented on the paper.

## Conflict of interest

The authors declare no conflict of interest.

## Supporting information

As a service to our authors and readers, this journal provides supporting information supplied by the authors. Such materials are peer reviewed and may be re‐organized for online delivery, but are not copy‐edited or typeset. Technical support issues arising from supporting information (other than missing files) should be addressed to the authors.

Supporting Information
