# Supplementary material for: Evaluation of an E. coli Cell Extract Prepared by Lysozyme‐Assisted Sonication via Gene Expression, Phage Assembly and Proteomics
Source: Chembiochem. 2021 Jul 29;22(18):2805–13. doi: 10.1002/cbic.202100257 (PMC8518995; doi:10.1002/cbic.202100257)
Supplement: Supplementary file 1 — Supporting Information [file CBIC-22-2805-s001.pdf]

ChemBioChem

Supporting Information

Evaluation of an *E. coli* Cell Extract Prepared by Lysozyme-Assisted Sonication via Gene Expression, Phage Assembly and Proteomics

Elisabeth Falgenhauer, Sophie von Schönberg, Chen Meng, Andrea Mückl, Kilian Vogele, Quirin Emslander, Christina Ludwig, and Friedrich C. Simmel*

Experimental Section

Chemicals. Unless otherwise noted all chemicals were ordered from Sigma Aldrich (exceptions are listed in Table S1). The composition of 2x YTP medium, S30A and S30B buffer was adopted from Sun et al.[1] The TXTL buffer containing amino acids, nucleotides, tRNAs and other ingredients was also prepared according to Sun et al. and screening experiments for Mg-glutamate, K-glutamate, PEG and DTT concentrations were performed (see Figure S2).

Cloning of plasmids. All plasmids were cloned using a standard restriction/ligation protocol. Linear DNA fragments were ordered from IDT and cloned into the target vector (pSB1A3). The final plasmid sequences are listed in Tables S6-S9. Primers were ordered from Eurofins Genomics. Plasmids were sequenced by GATC Services/Eurofins Genomics.

Bacterial strains and culture conditions. Figure 1 gives a quick and Figure S1 a detailed overview over the cell extract preparation workflow. Bacterial cell extracts were prepared from *E.coli* Rosetta 2 (DE3) cells. Cells from glycerol stocks were grown overnight in an incubator shaker (Innova44, New Brunswick) in 2x YT+P medium containing selective antibiotic (Chloramphenicol, Cm) at 37 °C and 250 rpm. 2x YT medium is very common for this purpose and is often supplemented with phosphate and also with glucose,[1-2] which avoids phosphatase induction and results in reduced ATP hydrolysis activity.[2a] On the following day, cells were diluted 1:100 in 2x YTP+Cm medium and cultivated either in eight shaking flasks (666 mL each, 37 °C, and 250 rpm) or in a 2 L bioreactor.

Cultivation of bacteria in a bioreactor. Cells were cultured in a 2 L lab scale bioreactor (Minifors 2, Infors) with pO2 monitoring and pH control. Initially, the culture was agitated at 500 rpm and aerated with pressurized air at a rate of 2 L/min. To keep the oxygen saturation over 14%, we regulated the aeration rate stepwise up to 4 L/min and increased the stirrer speed up to 1000 rpm. We used a constant feeding with a glucose solution at a rate of 0.85 g/(L h) during the entire cultivation time. As we grow the bacteria in 2x YT medium supplemented with potassium phosphate (monobasic and dibasic), the medium is buffered and the external pH control during the cultivation is not obligatory. Due to the favorable growth conditions in the bioreactor, the exponential growth phase was prolonged and we were thus able to harvest the cells in an OD range from 5-6. A linear fit in a semi-logarithmic plot revealed that the bacteria were still in the exponential growth phase at this point (see Figure S3). Growth in a bioreactor allowed us to increase the biomass yield (wet pellet) to 10 g per liter culture volume compared to shaking flask cultivation, which typically yielded only 2-2.5 g/L.

Cell harvest and washing. Cells were harvested at OD 1.8-2.0 when cultivated in shaking flasks, or at OD 5-6 when grown in a bioreactor. After harvesting, we distributed the cell suspension to four bottles (750 mL each) for centrifugation (15 min, 4 °C, max. speed 4600 rcf, Rotanta 460R, Hettich). In the case of shaking flask cultivation, a second round of cell harvesting was necessary. The supernatant was decanted and cells were resuspended in S30A buffer (300 mL per centrifuge bottle). Centrifugation and washing (2 bottles were pooled, washing with 2x 350 ml) was repeated. Afterwards, we resuspended the cell pellets in 2x 40 mL S30A buffer and transferred the suspension into two 50 mL falcon tubes. Cells were centrifuged at 3000 rcf at 4 °C for 10 min. The supernatant was decanted and the pellet was again centrifuged at 3000 rcf at 4 °C for 3 min. The supernatant was then removed using a pipette. After determination of the wet pellet mass (typical 20 g from bioreactor cultivation), the pellets were flash frozen in liquid nitrogen and stored at -80 °C.

Preparation of cell extract. Cell pellets obtained in the previous step were thawed on ice and resuspended in S30A buffer (1 mL buffer per gram pellet mass) by vortexing. The cell suspension was then split into 4 mL aliquots and up to 1 mg/mL lysozyme (Lysozyme from chicken egg, >40,000 units/mg, Sigma Aldrich) was added. After mixing by pipetting up and down the cell suspension was incubated on ice for 15 min to allow the lysozyme to degrade the peptidoglycan layer. The incubation on ice should prevent a loss in activity due to a proteolytic degradation of released proteins. Cells were sonicated on ice using a SONOPULS mini20 (Bandelin) with a working frequency of 30 kHz at 10% amplitude. We applied 0-20 pulse cycles with durations of 10 sec each. Tubes were sonicated in series, so the cooling time between different rounds is 10 sec times the number of tubes. Thus, our typical pausing time between sonication pulses was 80-100 sec (for 8-10 samples) per tube. Since throughput with sonication is limited, we recommend to not to prepare larger volumes than 40 mL cell suspension at a time (at a constant sample volume of 4 mL per tube). After every second cycle, samples were mixed by pipetting up and down using a 5 mL pipette with the tip cut off. After lysis, samples were transferred into 2 mL tubes and centrifuged at 20,000 rcf for 30 min - 60 min at 4 °C until a sufficiently stable pellet had formed. Pellet-free supernatant was transferred into 2 mL screw cap tubes (1-1.5 mL volume per tube), leaving the caps unscrewed. The open tube containing the cell extract was inserted in a 15 mL Falcon tube as described by Sun et al. [1] and incubated at 37 °C and 250 rpm for 80 min in an Innvova44 shaker for a run-off reaction. Afterwards, samples were transferred into individual reaction tubes and centrifuged at 12,000 rcf for 10 min at 4 °C. Pellet-free supernatant was transferred into 10 kDa MWCO dialysis tubing and dialyzed against S30B buffer at 4 °C for 3 h. Cell extract was then extracted from the tubing, distributed into 1.5 mL centrifugation tubes and centrifuged at 12,000 rcf for 10 min at 4 °C. Cell extract was finally

aliquoted into the desired aliquot size (usually 30 µl), flash frozen in liquid nitrogen and stored at -80 °C.

Bicinchoninic acid (BCA) assay. Each cell extract batch was subjected to a Bicinchoninic acid (BCA) assay (Pierce BCA Protein Assay Kit, Reducing Agent Compatible Thermo Fisher Scientific) according to the manufacturer's protocol to determine the total protein content of the prepared cell extracts. The results displayed in Figure 2A and C show the mean protein contents of three biological replicates, Figure S4 shows the protein content for each single extract.

Transcription-translation of fluorescent proteins (TXTL test). High copy number plasmids (iGEM part pSB1A3) containing a constitutive promoter (iGEM part J23106), an RBS (iGEM part B0034), the coding sequence for mScarlet-I (RFP), mVenus (YFP), GFPmut3 (iGEM part E0040) or mTurquoise-2 (CFP) and a Terminator (iGEM part B0015) were purified using a Qiagen Plasmid Midi Kit and afterwards phenol chloroform precipitated. Whereas Takahashi et al recommended to use a protein content of 10 mg/mL in the final cell-free protein expression reaction,[3] we used the same dilution factor for all tests independently of the protein content. Our samples contained 33.3% cell extract, 41.7% buffer solution and 25 % plasmid mix or water for blank samples. The final plasmid concentration in each sample was 3 nM and the TXTL buffer composition resulted in sample concentrations of 4 mM Mg-glutamate, 60 mM K-glutamate, 1.5 mM each amino acid except leucine, 1.25 mM leucine, 50 mM HEPES, 1.5 mM ATP and GTP, 0.9 mM CTP and UTP, 0.2 mg/mL tRNA, 0.26 mM CoA, 0.33 mM NAD, 0.75 mM cAMP, 0.068 mM folinic acid, 1 mM spermidine, 30 mM 3-PGA and 2.5% (w/v) PEG-8000.

Fluorescence acquisition. Transcription-translation of fluorescent proteins was monitored with a plate reader (FLUOstar Omega, BMG Lab Tech) at a temperature of 29 °C. Fluorescence measurements were performed every 3-6 min using the corresponding filter sets for RFP, YFP, GFP or CFP. Time traces were background corrected with blank values and molar concentrations were calculated using calibration curves for each of the four fluorescent proteins. To compare the fluorescence time traces, their maximum slopes (maximum protein expression rate) and end levels were determined. The mean end levels (mean of 3 biological replicates) are shown for the YFP reporter in Figure 2 as bar graphs for the single lysis settings. The data for RFP, GFP and CFP and the mean maximum protein expression rates (mean of 3 biological replicates) are shown in Figure S6.

Phage assembly. Phage assembly was performed according to the protocol of Rustad et al. [4] with the following adjustments: Phage DNA was mixed with cell extract, an energy solution and an amino acid solution as described in Sun et al. [1] using the same TXTL buffer composition as described in the TXTL test section.[5] For 6 reactions (of 13 µL volume

each), 2.5 μL PEG 8000 (36% w/v), 4 μL dNTPs (25 mM), 0.8 μL ATP (500 mM), 37.5 μL TXTL buffer, 2 μL GamS (150 μM), 28.5 μL cell extract and 1.6 μL DNA (10 nM) were mixed with nuclease-free water to a final volume of 80 μL. All constituents were mixed (except DNA) on ice and incubated for 5 min, followed by the addition of DNA. This 13-μL assembly mix was incubated for 4 h at 29 °C to express the bacteriophages.

Plaque assay. The plaque-assay was performed with the top-agar method, with 0.5% agarose in NZCYM (Carl Roth), a standard medium for *E. coli* cultures and bacteriophages.[6] The agar was melted and stored before use in a water bath at 48 °C. Separately, phage dilutions of 10^2-10^8-fold in phage buffer (1x PBS, 1mM $MgCl_2$, 1mM $MgSO_4$) were prepared. 100 μL of each dilution was mixed with an equal volume of an overnight culture of the corresponding host bacterium. This mixture was added to the 0.5% agarose NZCYM medium aliquots and poured on a 1% NZCYM agar plate. After solidified at room temperature, the plates were incubated at 37 °C until plaques became visible.

Sample preparation for mass spectrometry. All cell extracts were dried to completeness using a centrifugal evaporator (Centrivap Cold Trap -50, Labconco, US). The resulting pellets were dissolved in lysis buffer (8 M Urea, 5 mM EDTA, 100 mM NH_4HCO_3) to a final concentration of 1 mg/mL. Next, 45 μg of each sample was reduced with 10 mM dithiothreitol (DTT) (30 min at 30 °C) and alkylated with 55 mM 2-chloroacetamide (CAA) (30 min in the dark at 25 °C). The samples were diluted 1:4 in 50 mM NH_4HCO_3 and double-digested with trypsin (1 h and 13 h at 30 °C, Trypsin gold Mass Spectrometry Grade, Promega), which was added twice at a ratio of trypsin:protein = 1:100 (by mass). The reaction was stopped with 1% formic acid (FA) and the resulting peptides were purified. For that in-house built C18 tips (5 disks of Sep-Pak Vac C18 material, Waters, US) were equilibrated with 250 µl 100% acetonitrile (ACN), 250 µl elution solution (40% ACN, 0.1% FA) and 250 µl washing solution (2% ACN, 0.1% FA) at 1500 g. The samples were loaded into the tips (centrifugation for 2 min at 500 g) and washed three times with washing solution for 2 min at 1500 rcf. Finally, the peptides were eluted with 100 µl elution solution for 2 min at 500g. The samples were dried to completeness and resuspended in washing solution 45 µl right before the MS measurement.

Proteomics data acquisition. Generated peptides were analyzed on an Dionex Ultimate 3000 RSLCnano system coupled to an Orbitrap Fusion Lumos Tribrid Mass Spectrometer (Thermo Fisher Scientific, Bremen, GER). For each analysis an injection amount of ≈ 0.1 µg of peptides was delivered to a trap column (ReproSil-pur C18-AQ, 5 µm, Dr. Maisch, 20 mm x 75 µm, self-packed) at a flow rate of 5 µL/min in 100% solvent A (0.1% formic acid in HPLC grade water). After 10 min of loading, peptides were transferred to an analytical column (ReproSil Gold C18-AQ, 3 µm, Dr. Maisch, 400 mm x 75 µm, self-packed)

and separated using a 50 min gradient from 4% to 32% of solvent B (0.1% formic acid in acetonitrile and 5% (v/v) DMSO) at 300 nL/min flow rate. Both nanoLC solvents contained 5% (v/v) DMSO. The Fusion Lumos Tribrid Mass Spectrometer mass spectrometer was operated in data dependent acquisition and positive ionization mode. MS1 spectra (360–1300 m/z) were recorded at a resolution of 60,000 using an automatic gain control (AGC) target value of 4×10^5 and maximum injection time (maxIT) of 50 ms. After peptide fragmentation using higher energy collision induced dissociation (HCD), MS2 spectra of up to 20 precursor peptides were acquired at a resolution of 15,000 with an automatic gain control (AGC) target value of 5×10^4 and maximum injection time (maxIT) of 22 ms. The precursor isolation window width was set to 1.3 m/z and normalized collision energy to 30%. Dynamic exclusion was enabled with 20 s exclusion time (mass tolerance +/-10 ppm). MS/MS spectra of species that were singly-charged, unassigned or with charge states > 6+ were excluded.

Proteomics data analysis. Peptide identification and quantification was performed using the software MaxQuant (version 1.6.3.4) with its built-in search engine Andromeda.[7] MS2 spectra were searched against the *E. coli* (strain B / BL21-DE3) reference proteome from Uniprot (UP000002032, 4156 protein entries), supplemented with common contaminants (built-in option in MaxQuant). Trypsin/P was specified as proteolytic enzyme. Precursor tolerance was set to 4.5 ppm, and fragment ion tolerance to 20 ppm. Results were adjusted to 1% false discovery rate (FDR) on peptide spectrum match (PSM) level and protein level employing a target-decoy approach using reversed protein sequences. The minimal peptide length was defined as 7 amino acids, the "match-between-run" function was disabled. For full proteome analyses carbamidomethylated cysteine was set as fixed modification and oxidation of methionine and N-terminal protein acetylation as variable modifications.

Proteins were quantified using Label Free Quantification (maxLFQ [8]). The maxLFQ intensity was log transformed before downstream analysis. T-tests were used in the differential analysis (using R version 3.6.3). The false discovery rates (FDRs) were calculated from the p-value using the Benjamini-Hochberg method.[9] Proteins with FDR < 0.05 and unique proteins (proteins which were present in all three replicates of one sample, but not present in one of the three replicates of the other sample) were selected as significantly differentially expressed proteins and passed to the DAVID functional annotation[10] for enrichment analysis. To simplify the presentation, proteins in GO terms with FDR below 0.05 were roughly classified according to keywords using the UniProt database [11] related to protein expression and energy regeneration and their influence on gene expression was interpreted accordingly.

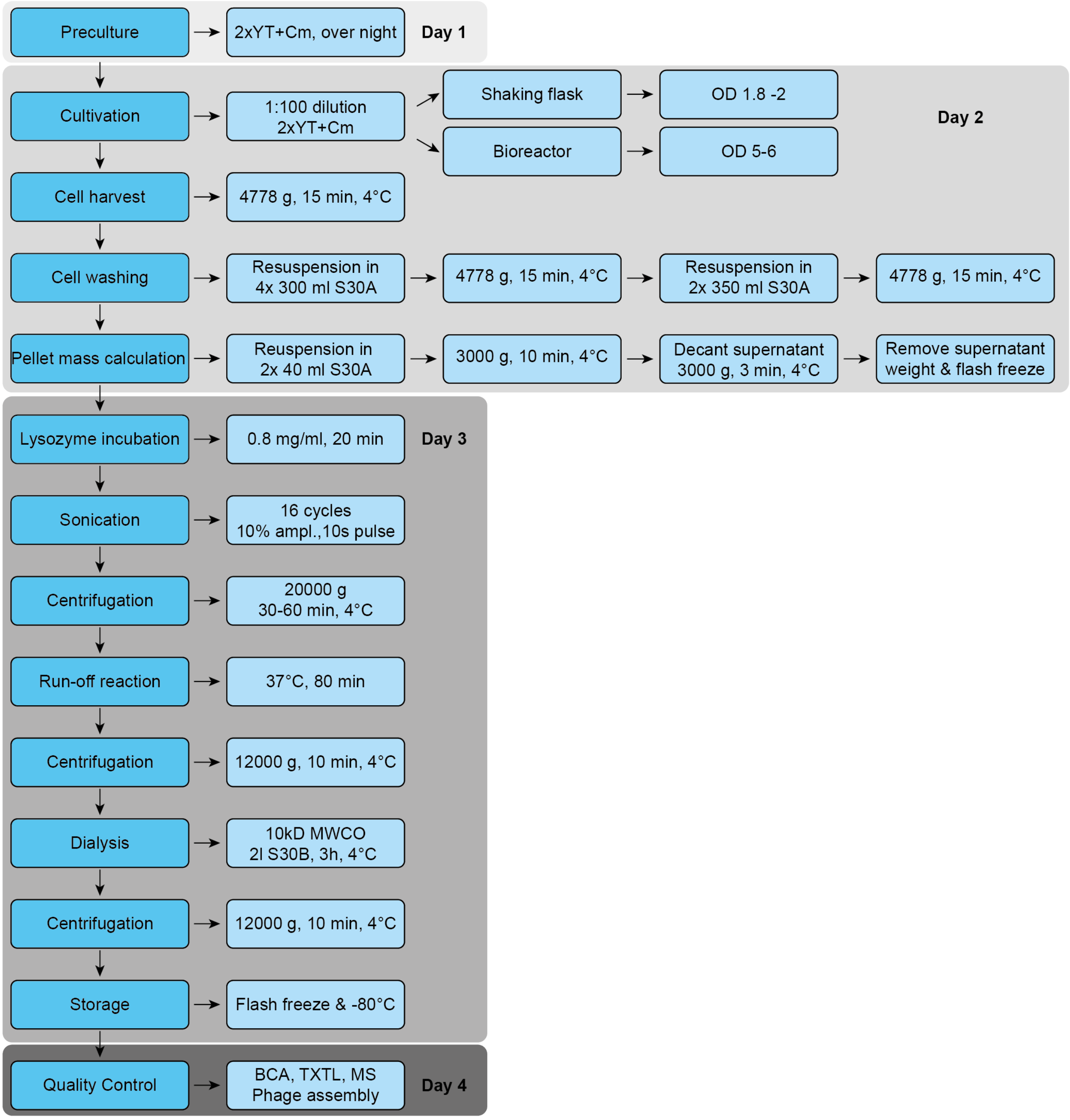

Figure S1: Detailed flow chart of the cell extract preparation protocol. See methods part in main text for more information.

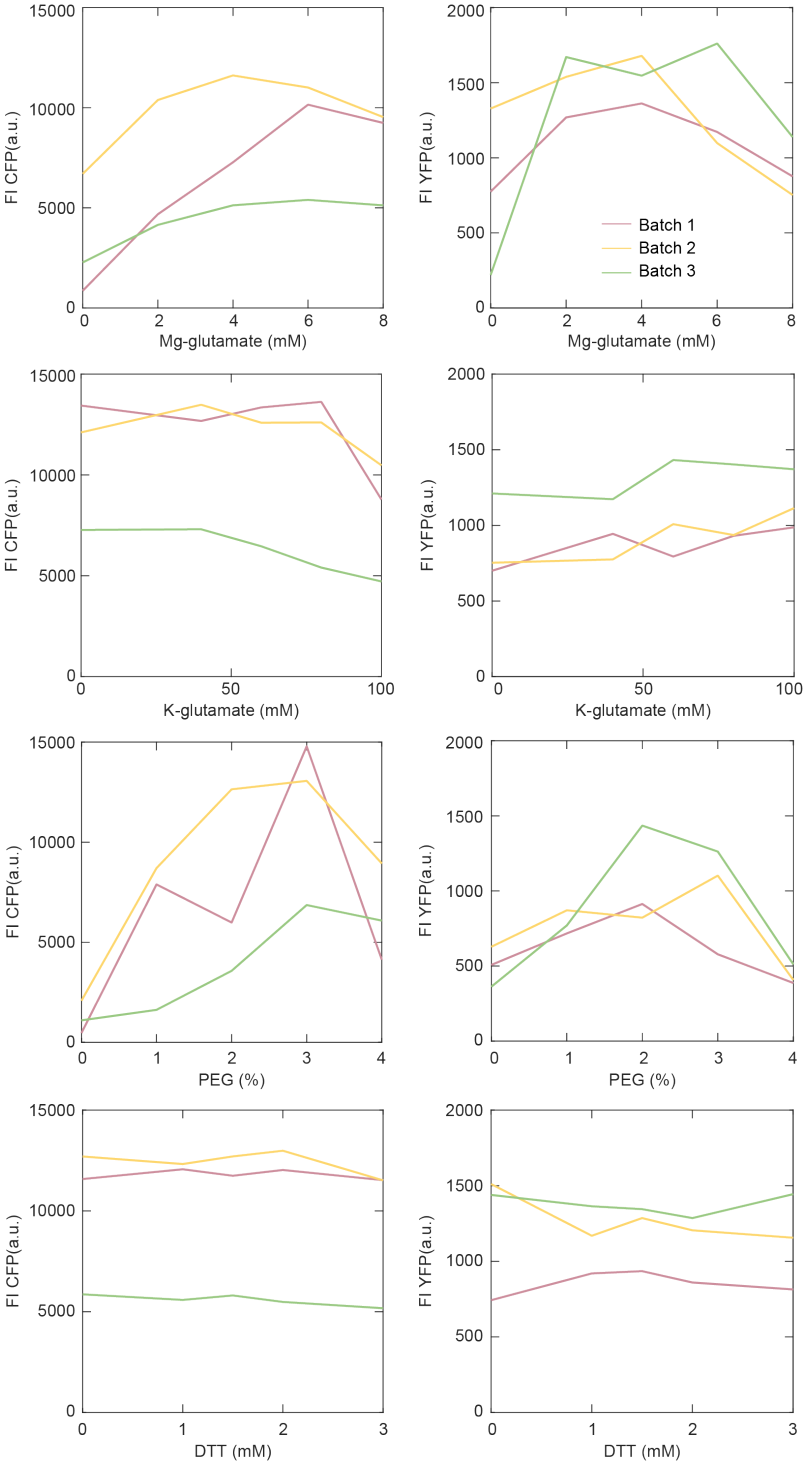

Figure S2: Cell extract buffer screening experiments. Mg-glutamate, K-glutamate, PEG and DTT concentration was screened for 3 different cell extract batches prepared using the S16/L0.8 lysis setting. The fluorescence end levels for 2 different reporter plasmids (CFP and YFP) are shown. 4 mM Mg-glutamate, 60 mm K-glutamate, 2.5% PEG and 0 mM DTT were chosen.

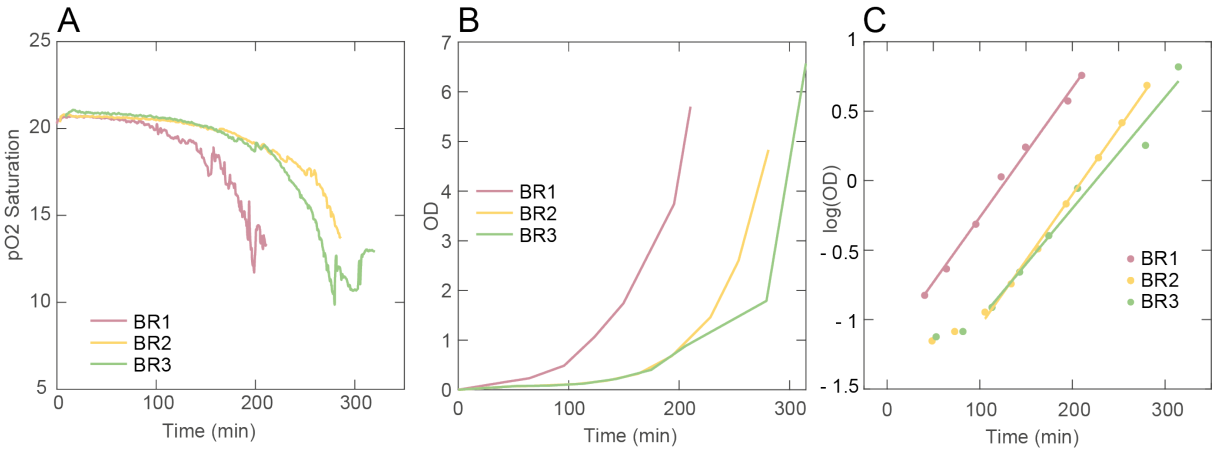

Batch	Slope (10⁻⁴ 1/min)	Doubling Time (min)	k (1/h)
BR1	93 ± 3	32.3 ± 0.3	1.29 ± 0.04
BR2	95 ± 2	31.8 ± 0.2	1.31 ± 0.03
BR3	80 ± 7	37.7 ± 0.9	1.10 ± 0.09

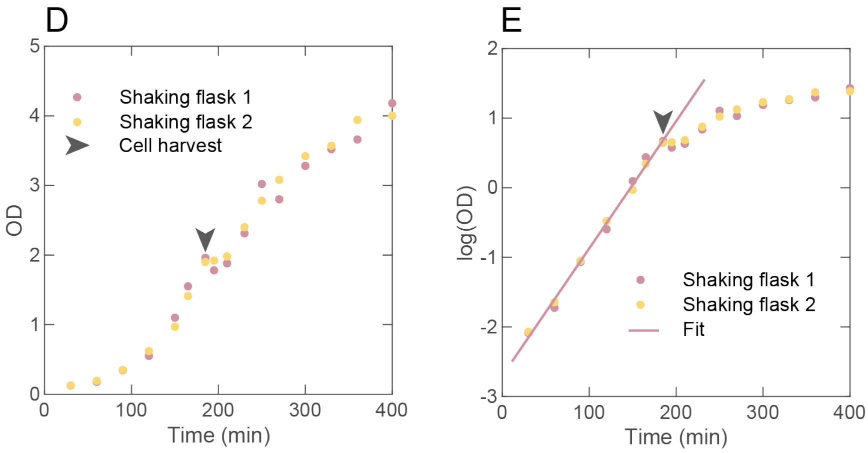

Batch	Slope (10^{-4} 1/min)	Doubling Time (min)	k (1/h)
Shaking Flask	183± 4	16.4 ± 0.1	2.53 ± 0.06

Figure S3: Growth curves for cultivation in shaking flasks and in a bioreactor. (A) pO_2-saturation plot. We fed glucose with a constant rate of 0.85 g/(l*h) and kept the oxygen level above 14% during the whole batch time by regulating the stirrer speed (500-1000 rpm) and the aeration rate (2-4 l/min) up. (B) OD was measured about every 30 min. Batches BR2 and BR3 show an elongated lag phase at the beginning. The desired OD was reached after about 200 min for batch BR1 and after about 280-300 min for batches BR2 and BR3. (C) Semi-logarithmic plot. The bacteria have doubling times between 32 and 38 min. (D) Growth curve for two different shaking flasks, cells are usually harvested between OD 1.8 and 2. (E) Semi-logarithmic plot. Bacteria were harvested in the late-log growth phase.

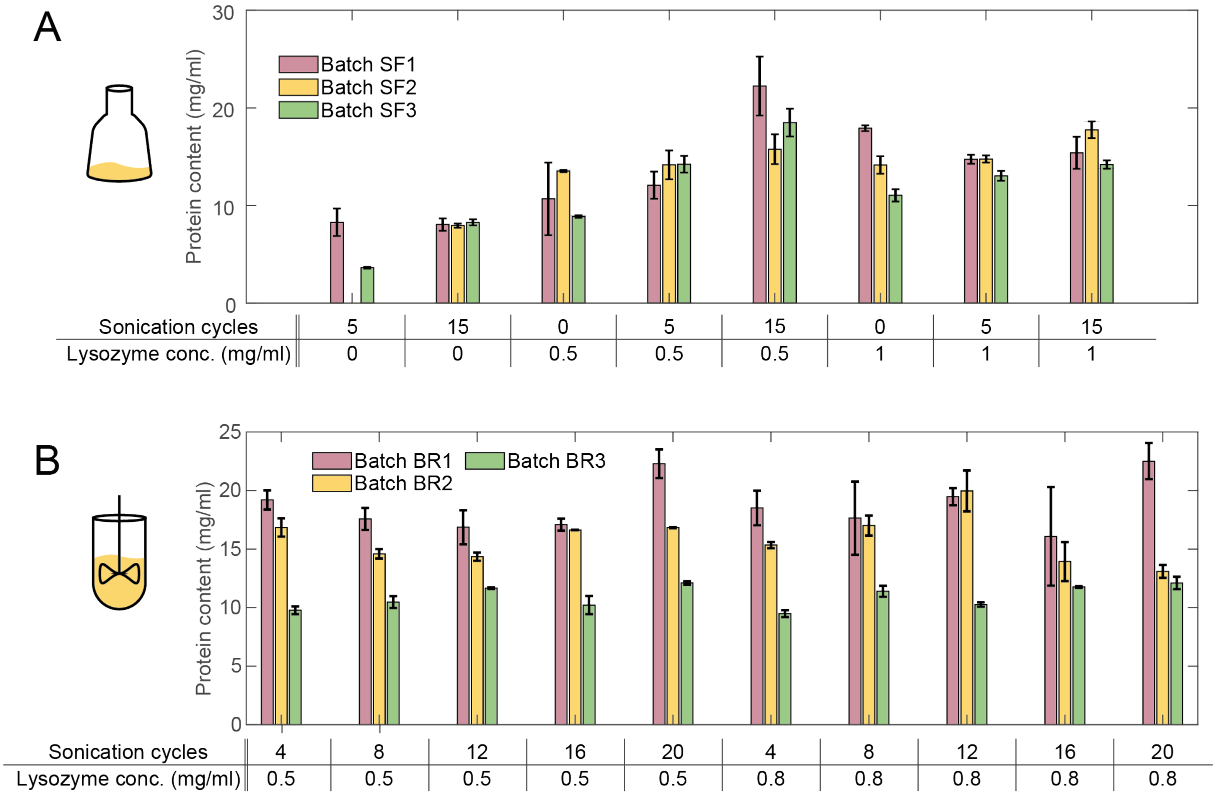

Figure S4: (A) For the shaking flask replicates lysozyme incubation had a higher impact on cell lysis than sonication cycles. The protein content was generally higher in samples without sonication cycles (S0/L0.5 and S0/L1) than in samples not incubated with lysozyme (S5/L0 and S15/L0). Samples which were treated with a combination of lysozyme incubation and sonication cycles showed a high protein content. (B) In contrast to the shaking flask replicates, the bioreactor replicates showed a higher deviation in protein content among the biological replicates than among the different lysis settings.

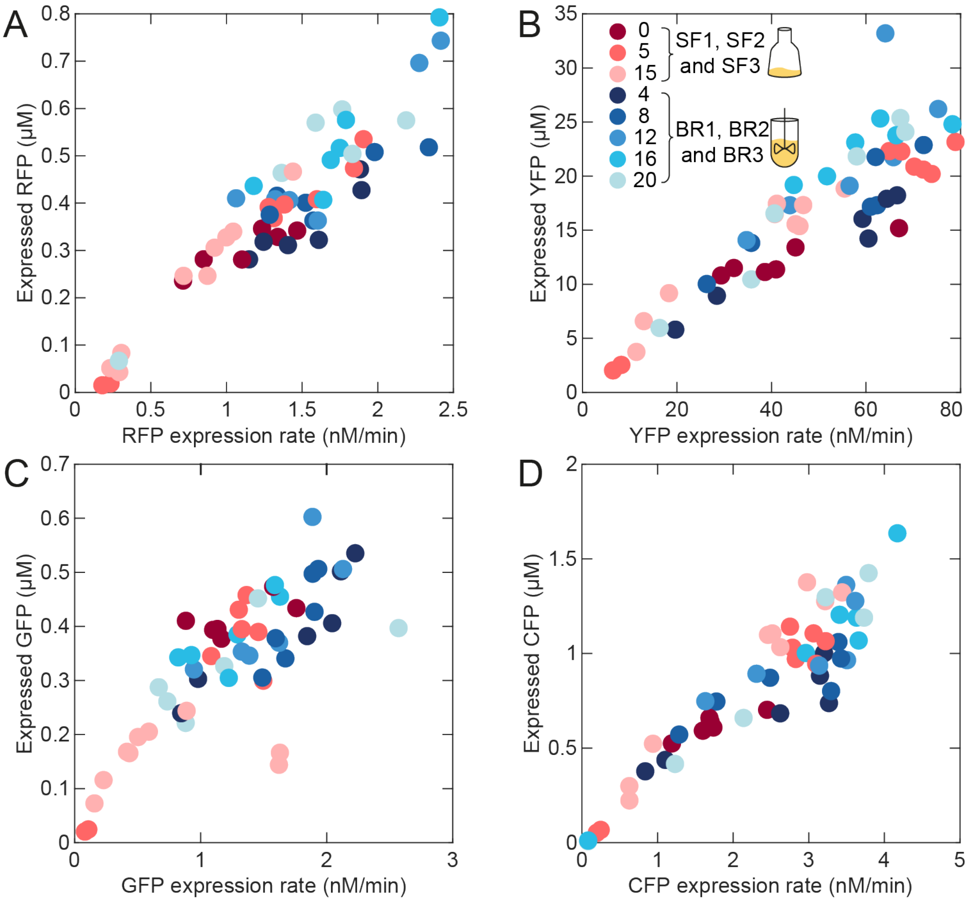

Figure S5: Expressed protein end level against maximum protein expression rates. The maximum expression rates correlates well with the expressed protein end levels.

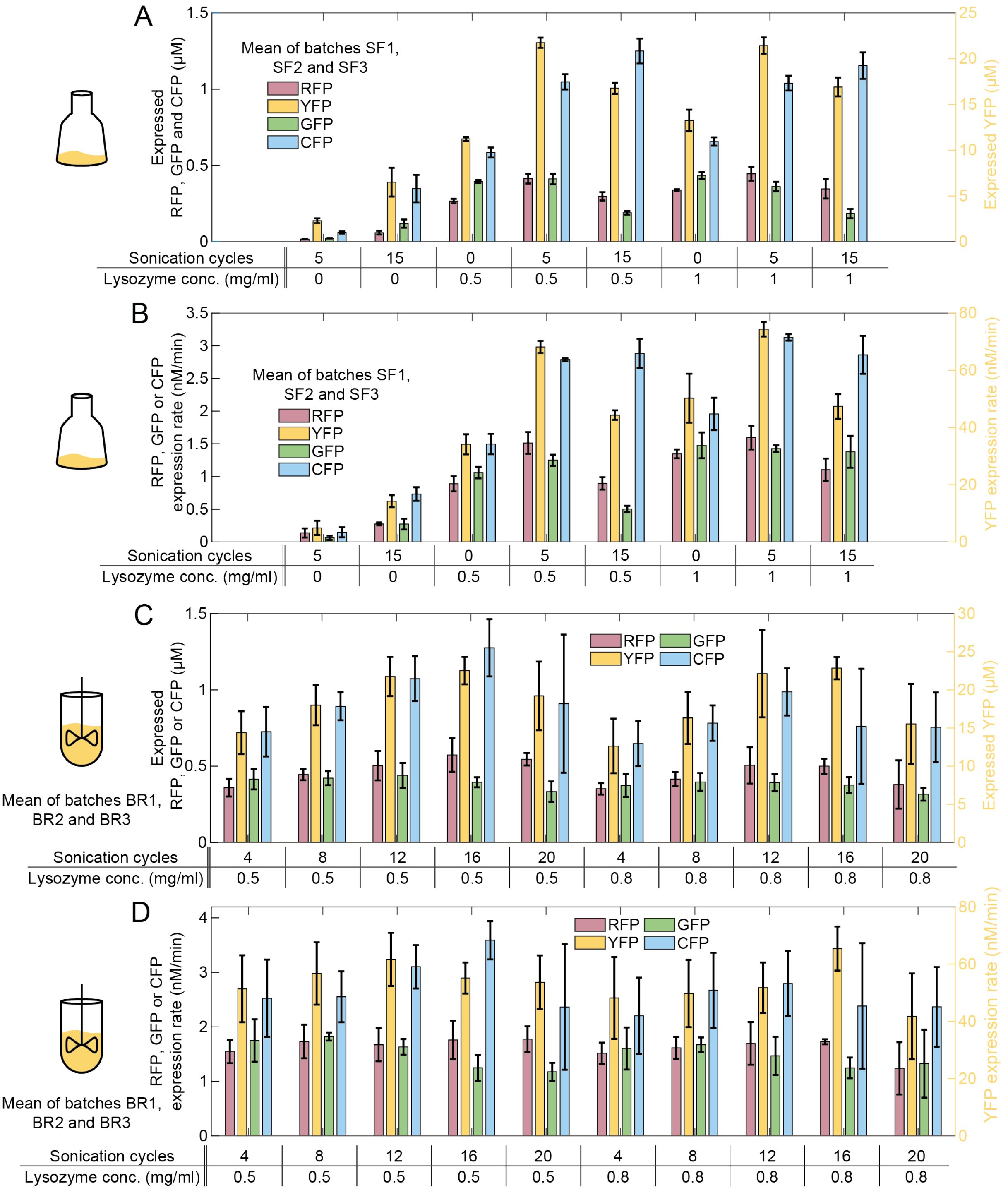

Figure S6: End levels of expressed proteins and maximum protein expression rates. (A) Shaking flask samples, which were not treated with lysozyme but with 5 or 15 sonication cycles, show the lowest fluorescence end levels in a cell-free test for all tested reporter proteins. Samples which were lysed without sonication cycles show higher signals, so lysozyme has no negative effect on the protein synthesis. The fluorescence intensities of samples with nonzero lysis conditions are the highest, a signal decrease can be observed for 15 sonication cycles. (B) The maximum protein expression rates show the same trends. (C) For both lysozyme concentrations an increase of sonication cycles results in an increased fluorescence signal in the bioreactor samples. Independent of the lysozyme concentration, 12-16 sonication cycles appear optimal, while 20 cycles result in reduced signals. (D) Mean of bioreactor replicates BR1, BR2 and BR3. The maximum expression rate correlates with the expressed protein end levels.

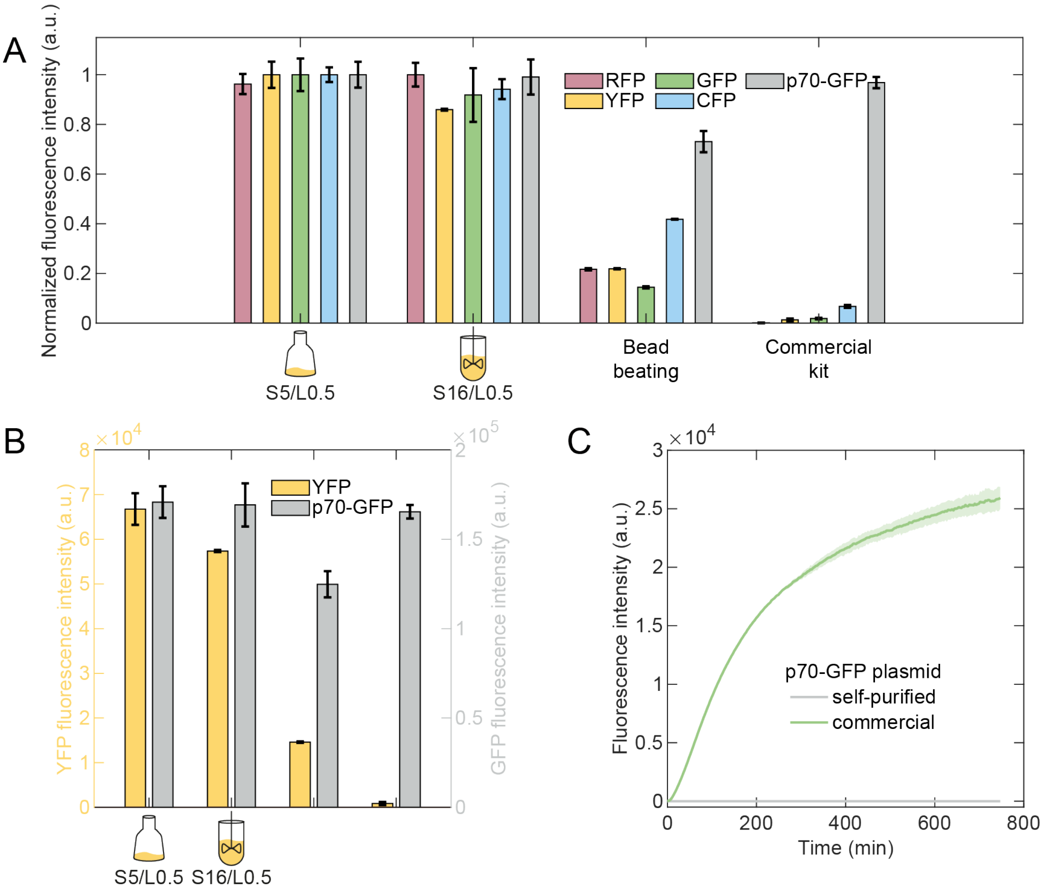

Figure S7: Comparison of the best shaking flask replicates with the best bioreactor replicates, a bead beating batch and a commercial cell extract. (A) Normalized fluorescence data for all tested reporters. The commercial extract shows low levels for all reporter plasmids except the p70 control plasmid. (B) Not normalized fluorescence data for the YFP plasmid and the GFP control plasmid. The levels are very different, but these levels depend not just on protein concentration but also on quantum yield, brightness and other parameters. (C) p70-GFP control plasmid in commercial cell extract. We purified the p70-GFP control plasmid using our standard technique and performed a TXTL test. The commercial plasmid has a high signal whereas our self-purified one has almost no signal. The commercial cell extracts seems to be sensitive to residual chemicals in our plasmid.

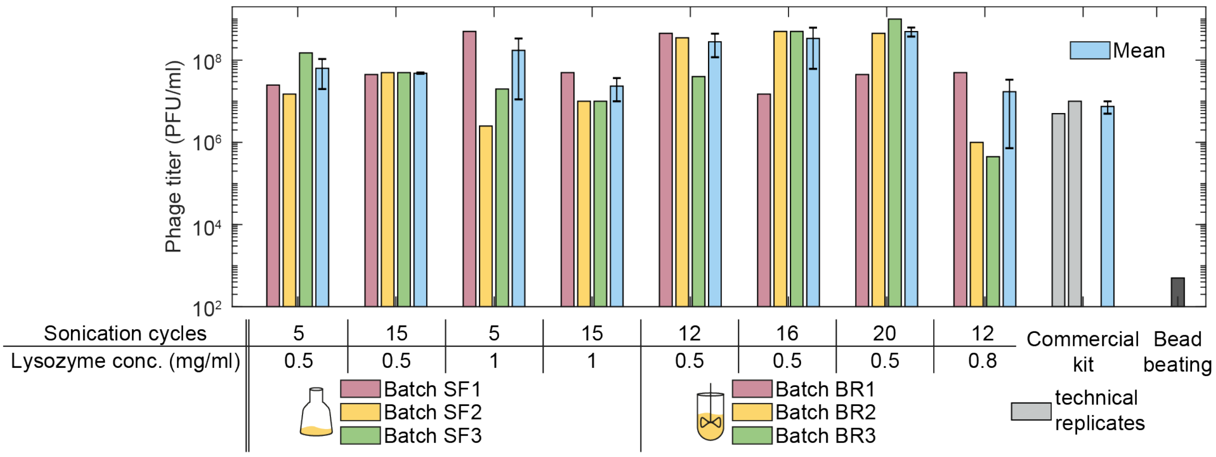

Figure S8: Plaque assays for the shaking flask and bioreactor replicates. For all self-made cell extracts except for the S12/L0.8 samples comparable phage titers were measured. For the commercial kit one order of magnitude less phages could be assembled and the bead beating batch performed much worse, just 500 phages could be counted in one replicate, no plaques could be counted for the second replicate.

A

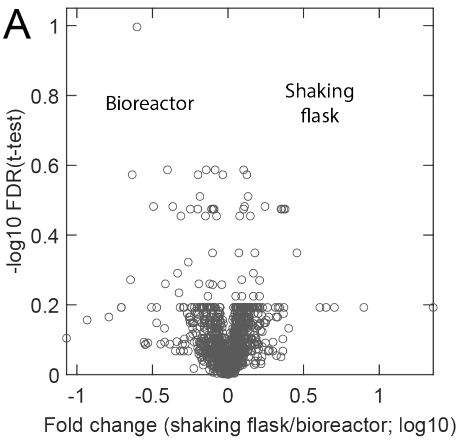

B

	shaking flask	bio-reactor
Total number of proteins	1480	1536
Unique proteins	65	0
Thereof n=3 n=0	11	0
Proteins with FDR <0.05	0	8
Proteins in GO with FDR <0.05	3	0
Thereof n=3 n=0	3	0
Proteins in GO with 0.05 <FDR <0.2	0	0
Thereof n=3 n=0	0	0

C

Uniprot ID	Protein name	Gene name
A0A140N619_ECOBD	Glycerol-3-phosphate dehydrogenase	**glpA** ECBD_1419, HO396_11015
A0A140N9S7_ECOBD	Anaerobic glycerol-3-phosphate dehy...	**glpB** ECBD_1418, HO396_11020
A0A140NA34_ECOBD	Anaerobic glycerol-3-phosphate dehy...	**glpC** ECBD_1417, HO396_11025

Figure S9: Comparing proteomes of extracts from shaking flask and bioreactor. (A) Volcano plot of shaking flask samples S5/L0.5 against bioreactor samples S5/L0.5. No protein had a FDR smaller than 0.05, but some unique proteins were found and used for enrichment analysis. (B) Summary of protein numbers. Compared to the bioreactor samples (S5/L0.5) the shaking flask extracts (S5/L0.5) contain 11 unique proteins, which were subjected to an enrichment analysis. (C) Result of enrichment analysis. For the shaking flask samples just 3 proteins were found in GO terms with GO FDR < 0.05. These proteins can be assigned to anaerobic growth conditions. In summary shaking flask extracts and bioreactor extracts, which were treated with the same lysis conditions show no differences in their proteome except for abundance of proteins related to anaerobic growth. This is an expected result as we in contrast to bioreactor cultivation did not provide additional oxygen in shaking flask cultivation.

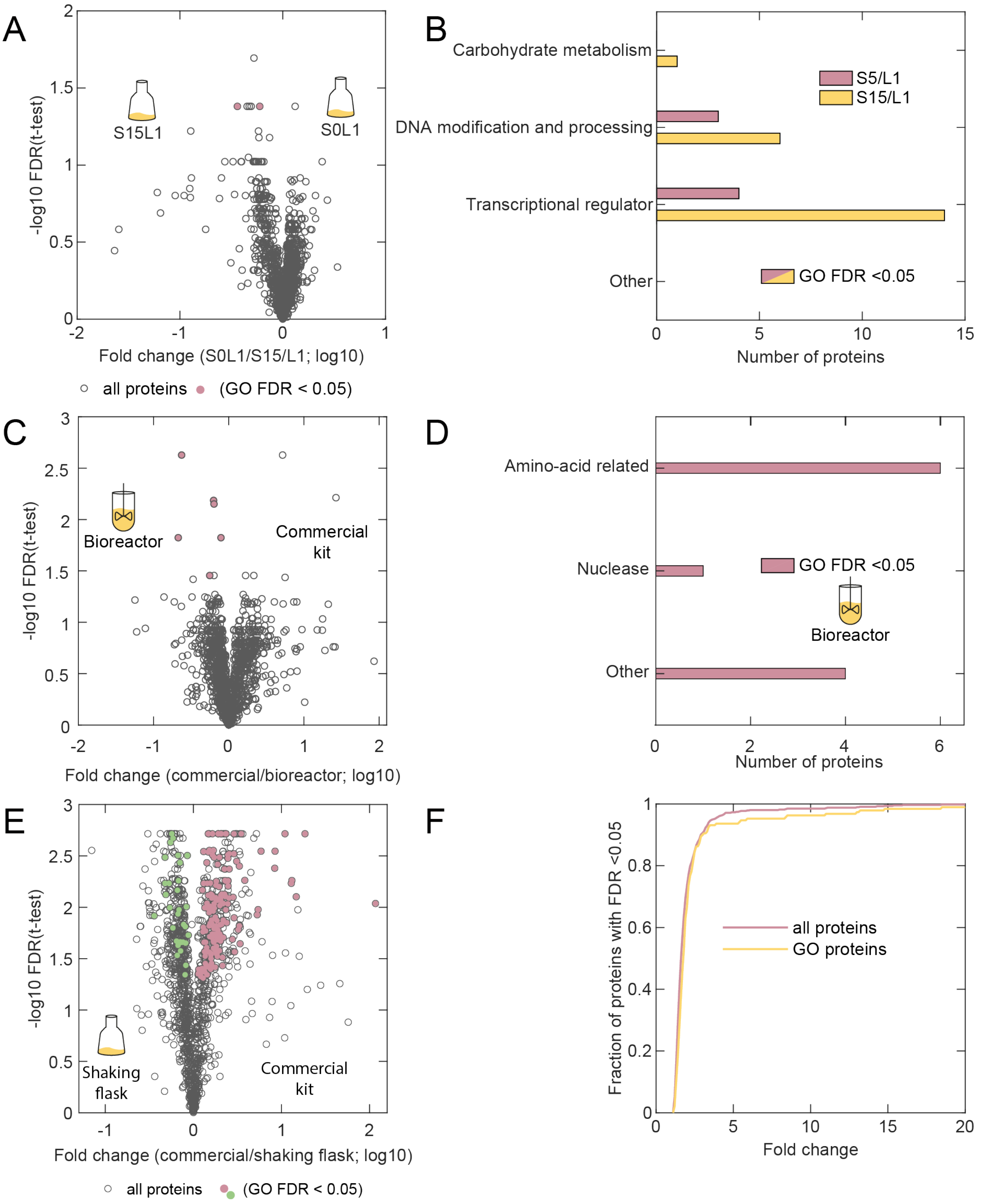

Figure S10: Comparison of the proteomes of extracts prepared from bioreactor and shaking flask cultures and a commercial kit. (A) Volcano plot of shaking flask samples S0/L1 against S15/L1. The t-test of S0/L1 against S15/L1 extracts gives 1 and 6 proteins with FDR < 0.05 (i.e., –log10(FDR) > 1.3). In addition, 3 (S0/L1) and 47 (S15/L1) unique proteins could be found and were also subjected to an enrichment analysis. (B) Enrichment analysis derived from the comparison of S0/L1 against S0/L5 and from the comparison shown in (A). No GO terms with FDR<0.05 could be found for the S0/L1 extracts in both comparisons but in total 7 (S0/L1 against S5/L1) and 21 (S0/L1 against S15/L1) proteins were found in GO terms with FDR < 0.05 for the S5/L1 and S15/L1 samples respectively. For both cases these could be assigned to keywords related to DNA replication, relaxation, repair or recombination or were transcriptional regulators. (C) Volcano plot of bioreactor samples S5/L0.5 against the commercial kit. The commercial and bioreactor extracts differ in abundance of just 8 and 5 proteins with FDR

< 0.05 (i.e., –log10(FDR) > 1.3), but had 30 and 121 unique proteins, which were subjected to an enrichment analysis. (D) Enrichment analysis derived from the comparison shown in (C). GO terms with FDR<0.05 included in total 11 proteins in the bioreactor samples, which were related in amino-acid biosynthesis, a nuclease or not relevant for cell-free gene expression. (E) Volcano plot of shaking flask samples S5/L0.5 against the commercial kit. The commercial and shaking flask extracts differ in abundance of 309 versus 356 proteins with FDR < 0.05 (i.e., –log10(FDR) > 1.3). Proteins with an FDR below 0.05 and proteins exclusively found only in shaking flask preparations or in commercial extract were subjected to an enrichment analysis. Proteins which were assigned to GO terms with an FDR below 0.05 are highlighted in the plot. (F) Fraction of proteins with FDR<0.05 against fold change and fraction of proteins found in GO terms with FDR<0.05 against fold change (unique proteins are not considered, as no fold change can be calculated). 27% of the proteins are less than 1.5 fold enriched, 67% less than 2 fold and 90% less than 3 fold.

Proteomic differences between our shaking flask replicates SF1, SF2 and SF3 with lysis setting S5/L0.5 and the commercial extract

The proteomics analysis revealed the greatest differences between our shaking flask batches S5/L0.5 and the commercial kit. The differences can be a result of differences in the culture conditions, the lysis procedure and other cell extract processing steps. Even though we had no detailed information about the preparation procedure of the commercial kit, we compared the two extracts, as the GFP expression yield was comparable. We found in total 172 (commercial extract, thereof were 15 unique proteins) and 31 (shaking flask batches, thereof were no unique proteins) proteins in GO terms with FDR<0.05, thereof were 90% of the proteins less than 3 fold enriched (67% less than 2 fold and 27% less than 1.5 fold enriched, see Figure S10F). The potential role of the single proteins is discussed in the following:

Energy metabolism. Compared to commercial extract, in our home-made batches 9 out of 10 enzymes from the glycolysis pathway were enriched, including glucokinase, which converts cytosolic glucose into glucose-6-phosphate (triosephosphate isomerase was the only enzyme that was not present at a higher abundance). Further, we found enzymes such as adenylate kinase or guanylate kinase enriched in our self-made batches, which potentially play a role in ATP and GTP regeneration *in vitro*. On the other hand, we detected higher abundance of the subunits IIB and IIC of *E. coli's* major transmembrane carbohydrate transport system (the Pst system) in the commercial extract. This indicates that the lysis conditions used for the commercial extract might cause a higher fragmentation of the membrane and thus a more efficient release of trans-membrane proteins. In contrast to glycolysis, enzymes of the TCA cycle were enriched in the commercial extract. In shaking flask batches, we also detected an increased amount of dehydrogenases encoded by the *glpABC* operon, which belongs to the glycerol kinase pathway and is responsible for anaerobic energy generation. No differences were detected in the pentose phosphate pathway.

Transcription/translation. Together with core RNA polymerase, sigma factor RpoD (σ^{70}) dominates the transcription in exponentially growing cells. We found RpoD more highly abundant in the commercial extract. Furthermore, during envelope stress several sigma factors are upregulated in *E. coli,* including sigma factor RpoE (σ^{24}), which was also found more abundant in the commercial extract. Other stress factors such as translational regulator CsrA (envelope/periplasmic stress) and nitrogen-limitation factor RpoN (σ^{54}) were enriched in the commercial cell-free system, as well as the ribosomal subunit S22, which is associated with stationary bacterial growth. Apart from these stress indicators, a variety of other transcription regulators such as LacI, MarR, GntR, DeoR or LysR were enriched compared to our home-made shaking flask batches.

In addition, also translational capacity appeared to be enriched in the commercial system. In particular, we found 20 out of the 22 ribosomal proteins of the 30S subunit at higher abundance in the commercial extract, including S22 (see above) and the essential ribosomal protein S12, which takes part in both tRNA and ribosomal subunit interactions. In case of the 50S subunit, we found 24 out of 33 ribosomal proteins more abundant in the commercial extract, including the small ribosomal protein L34 (5.3 kDa). Also other translation-related proteins showed higher abundance in the commercial

extract such as initiation (IF-2, IF-3) and elongation (EF-4, EF-Tu, SelB) factors, but also the ribosomal silencing factor (RsfS) which inhibits ribosome association and prevents translation.

Degradation of nucleic acids and proteins. We also found notable differences between the cell extracts in degradation pathways. In the commercial extract ribonucleases 2 and E were enriched (p-value 0.05), which are mainly involved in mRNA degradation. Other ribonucleases participating in RNA maturation and processing (RNAse 3, G, PH and R) were also more abundant in the commercial extract. On the other hand, endoribonuclease L-PSP, also acting on mRNA, was found more highly concentrated in self-made batches. We also investigated the presence of proteases in the cell extracts. We found both subunits HslV and HslU (annotation at the transcript level) of the proteasome-like degradation complex HslVU (ClpQY) enriched in the commercial extract, which unfolds proteins under ATP consumption.

Biosynthesis. Interestingly, many proteins involved in amino acid biosynthesis were more abundant in our self-made CF system compared to the commercial extract, suggesting their potential use for amino acid production inside the extract starting from inexpensive precursors. Finally, the chaperone cofactor GroES was more highly expressed in the commercial batch, but not its chaperone complex GroEL, even though it is encoded by the same operon.

Table S1: List of chemicals which were not ordered from Sigma Aldrich.

2-Chloroacetamide (CAA)	Merck (GER)
Acetonitrile (ACN)	Merck (GER)
CTP	Carl Roth (GER)
GamS	Arbor Bioscience (US)
Glycerol	Carl Roth (GER)
GTP	Carl Roth (GER)
IPTG	Carl Roth (GER)
Nuclease-free water	Carl Roth (GER)
Phosphate buffered saline (PBS)	VWR Life Science (GER)
RTS Amino Acid Sampler	Biozym Scientific (GER)
Tris	Carl Roth (GER)
Trypsin	Roche (CH)
UTP	Carl Roth (GER)

Table S2: All buffer and media compositions were adapted from Sun et al. [1]. Anyway the composition of the growth medium and the buffers needed for cell washing and cell extract dialysis are listed below. A detailed protocol for the TXTL buffer preparation is shown in the protocol of Sun et al.[1].

a) 2xYTP medium

2xYT	31 g/l
K_2HPO_4	40 mM
KH_2PO_4	22 mM

b) S30A (cell washing, 2l are needed)

Potassium glutamate	60 mM
Magnesium glutamate	14 mM
Tris	50 mM

To reach pH 7.7, titrate with acetic acid. Add DTT to 2mM final concentration just before use. Store at 4 °C.

c) S30B (cell extract dialysis, 2l are needed)

Potassium glutamate	60 mM
Magnesium glutamate	14 mM
Tris	5 mM

To reach pH 8.2, titrate with 2 M Tris. Add DTT to 1 mM final concentration just before use. Store at 4 °C.

Table S3: Characteristics of reporter proteins. Codon adaption indices for mScarlet, mVenus, GFP and mTurquoise calculated by CAIcal.[12] The codon usage table of Escherichia coli B and of Shigella flex. 2a were used for the calculation. The second one is more related to MRE600, which is the origin strain of the purified tRNA used for the TXTL buffer. The translation rate was predicted using an RBS calculator.[13]

**Protein**	**mScarlet I** [14]	**mVenus** [15]	**GFP mut3** [16]	**mTurquoise 2** [17]	**p70-GFP (deGFP3)** [18]
Ex. Max (nm)	569	515	500	434	508
Em. Max (nm)	593	527	513	474	518
QY	0.54	0.64	0.39	0.93	0.19
Brightness	56.16	66.56	34.87	27.9	5.07
maturation time (min)	36	17.6	4.1	33.5	
pKa	5.4	5.5		3.1	6.9
additional mutations	none	2V--> QK; 70Q-->M; 81Q-->R; 232L-->H	2S-->R	147I-->F	2SKGE-->---; 64F-->L; 203C-->T
CAI E. coli B	0.636	0.878	0.588	0.782	0.646
CAI Shigella flex. 2a	0.765	0.934	0.733	0.853	0.791
GC content full mRNA	49.31	47.6	40.9	50.11	59.13
GC content CDS	49.5	47.3	39.05	50.56	61.06
Predicted translation rate	3052	905	746	357	9373
ΔG total	-2.01	0.69	1.12	2.76	-4.51
ΔG mRNA-rRNA	-7.53	-7.53	-9.42	-10.08	-10.1
ΔG spacing	0.67	0.67	0.67	0.67	0.29
ΔG stacking	0	0	0	0	0
ΔG standby	4.9	4.9	4.9	4.9	0.01
ΔG start	-2.76	-2.76	-2.76	-2.76	-2.76
ΔG mRNA	-2.79	-5.49	-7.81	-10.11	-8.33

Table S4: T-test for TXTL data. We aimed to proof our hypothesis, that our data show an optimum in the number of sonication cycles. The data shown in Figure 2 B and D were split in two sets with fixed lysozyme concentration and different sonication cycles: For shaking flask cell extracts these were 0, 5, and 15 sonication cycles in combination with a lysozyme concentration of 0.5 or 1 mg/ml respectively (data set 1 and 2; samples S5/L0 and S15/L0 were excluded from the t-test). For the bioreactor extracts these were 4, 8, 12, 16, and 20 sonication cycles in combination with a lysozyme concentration of 0.5 or 0.8 mg/ml respectively (data set 4 and 5). As we observe the same trend in the data independent on the lysozyme concentration, we also introduced combined data sets (data set 3 and 6). A parabola $y=a \cdot x^2+b \cdot x+c$ with x being the number of sonication cycles and y being the TXTL end level of the YFP reporter was fitted to the data sets. The fit parameter a was tested against the hypothesis 'Fit parameter a is zero' and the according p-value was calculated. We could reject the null hypothesis for all data sets (p-value < 0.05) except for data set 4, which has a p-value of 0.06. So the observed optima in the TXTL data are statistically significant.

Culture method	Lysozyme (mg/ml)	Data set	p-value
Shaking flask	0.5	1	$1.32 \cdot 10^{-5}$
	1	2	$1.20 \cdot 10^{-3}$
	0.5 and 1 combined	3	$1.60 \cdot 10^{-8}$
Bioreactor	0.5	4	$6.43 \cdot 10^{-2}$
	0.8	5	$4.46 \cdot 10^{-2}$
	0.5 and 0.8 combined	6	$8.30 \cdot 10^{-3}$

Table S5: Comparison of different cell extract preparation protocols.

	This study	Sun et al. [1]	Kwon and Jewett [2b]	Fujiwara and Doi [19]
Strain	Rosetta 2 (DE3)	Rosetta 2 (DE3)	BL21 star (DE3)	BL21(DE3) codon plus (RIL)
Culture method	Shaking flask or bioreactor	Shaking flask	Shaking flask or bioreactor	Shaking flask
Culture medium	2xYTP shaking flask; 2xYTGP bioreactor	2xYTP	2xYTPG	LB
IPTG induction	No	No	Tested, but usually not used	Yes
Lysis method	Lysozyme incubation + sonication	Bead beating	Sonication	Lysozyme incubation + osmotic shock + freeze thaw cycles
Cell extract processing steps (after lysis)	In total 3 centrifugation steps; run off reaction; dialysis	In total 3 centrifugation steps; run off reaction; dialysis	1 centrifugation step; (run-off reaction and second centrifugation step was also tested)	1 centrifugation step (buffer exchange was also tested)
Total protein content (mg/ml)	15	30	40	20-30
Expressed protein (mg/ml)	0.6	0.75	1	0.25-0.5
TXTL test conditions	J23106 promoter; 9 nM corresponds to 17 µg/ml plasmid concentration	Lamda promoter or T7 promoter	Addition of T7 polymerase; T7 promoter (13.3 µg/ml plasmid concentration)	T7 promoter (1,5 nM template concentration) or OR2OR1 (10nM plasmid concentration)

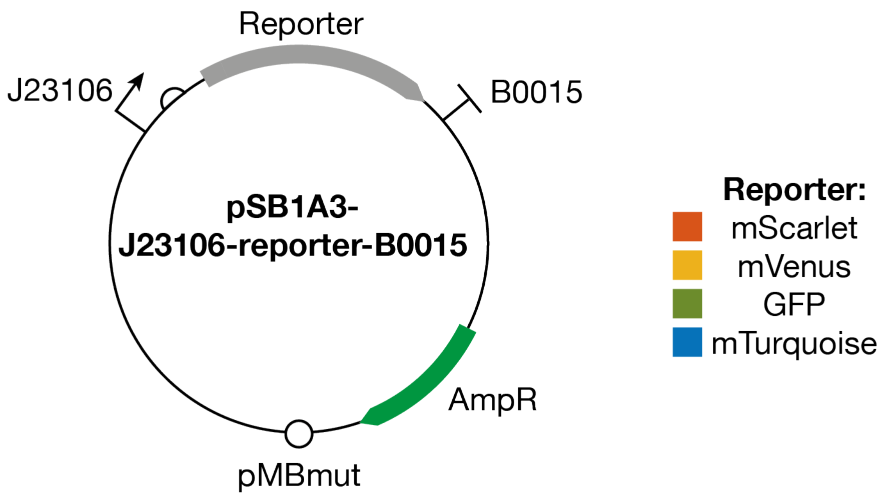

Figure S11: Plasmid maps for TXTL tests. A mScarlet, mVenus, E0040 GFP or mTurquoise reporter was cloned in a pSB1A3 backbone containing a J23106 promoter, a B0034 ribosome binding site (RBS) and a B0015 terminator.

Table S6: pSB1A3-J23106-B0034-mScarlet-B0015

cccctggaagctccctcgtgcgctctcctgttccgaccctgccgcttaccggatacctgtccgcctttctcccttcgggaagcgtggcgctttctcata
gctcacgctgtaggtatctcagttcggtgtaggtcgttcgctccaagctgggctgtgtgcacgaaccccccgttcagcccgaccgctgcgccttat
ccggtaactatcgtcttgagtccaacccggtaagacacgacttatcgccactggcagcagccactggtaacaggattagcagagcgaggtatg
taggcggtgctacagagttcttgaagtggtggcctaactacggctacactagaagaacagtatttggtatctgcgctctgctgaagccagttacctt
cggaaaaagagttggtagctcttgatccggcaaacaaaccaccgctggtagcggtggtttttttgtttgcaagcagcagattacgcgcagaaaa
aaaggatctcaagaagatcctttgatcttttctacggggtctgacgctcagtggaacgaaaactcacgttaagggattttggtcatgagattatcaa
aaaggatcttcacctagatccttttaaattaaaaatgaagttttaaatcaatctaaagtatatatgagtaaacttggtctgacagttaccaatgcttaa
tcagtgaggcacctatctcagcgatctgtctatttcgttcatccatagttgcctgactccccgtcgtgtagataactacgatacgggagggcttacca
tctggccccagtgctgcaatgataccgcgagacccacgctcaccggctccagatttatcagcaataaaccagccagccggaagggccgagc
gcagaagtggtcctgcaactttatccgcctccatccagtctattaattgttgccgggaagctagagtaagtagttcgccagttaatagtttgcgcaa
cgttgttgccattgctacaggcatcgtggtgtcacgctcgtcgtttggtatggcttcattcagctccggttcccaacgatcaaggcgagttacatgatc
ccccatgttgtgcaaaaaagcggttagctccttcggtcctccgatcgttgtcagaagtaagttggccgcagtgttatcactcatggttatggcagca
ctgcataattctcttactgtcatgccatccgtaagatgcttttctgtgactggtgagtactcaaccaagtcattctgagaatagtgtatgcggcgaccg
agttgctcttgcccggcgtcaatacgggataataccgcgccacatagcagaactttaaaagtgctcatcattggaaaacgttcttcggggcgaa
aactctcaaggatcttaccgctgttgagatccagttcgatataacccactcgtgcacccaactgatcttcagcatcttttactttcaccagcgtttctgg
gtgagcaaaaacaggaaggcaaaatgccgcaaaaaagggaataagggcgacacggaaatgttgaatactcatactcttcctttttcaatatta
ttgaagcatttatcagggttattgtctcatgagcggatacatatttgaatgtatttagaaaaataaacaaataggggttccgcgcacatttccccgaa
aagtgccacctgacgtctaagaaaccattattatcatgacattaacctataaaaataggcgtatcacgaggcagaatttcagataaaaaaaatc
cttagctttcgctaaggatgatttctggaattcgcggccgcttctagagtttacggctagctcagtcctaggtatagtgctagctactagagaaagag
gagaaactcgagatggtgtcaaagggagaggcggttatcaaggaatttatgcgctttaaagtccacatggagggcagcatgaacgggcacg
agtttgaaattgagggggagggggagggccgtccttatgaaggtactcagactgctaaactgaaggtgacaaaaggtggccccttgcctttctc
gtgggacatcctgtcgccacaattcatgtacgggagccgcgcctttatcaaacatcccgcagatattcctgattactataaacaatctttcccgga
aggtttcaaatgggaacgcgtcatgaattttgaggacgggggcgctgtcacagttactcaggacacctccttggaagacggcacattgatttaca
aggttaagttgcgcggcacaaacttcccccctgacgggccagtaatgcaaaagaaaactatgggttgggaggcgtctacagaacgtttatacc
ccgaagacggggtgctgaaaggtgacattaagatggccctgcgcctgaaggacggcggtcgctatcttgccgactttaaaactacttataagg
ctaaaaaaccagtccagatgccaggcgcctataatgttgaccgcaagttagacatcacctcacataatgaagactataccgttgtagaacaat
acgagcgcagcgagggtcgtcacagtaccgggggggatggatgaattatacaaataataacggatccgctgtccgccaggcatcaaataaaa
cgaaaggctcagtcgaaagactgggcctttcgttttatctgttgtttgtcggtgaacgctctctactagagtcacactggctcaccttcgggtgggcct
ttctgcgtttatatactagtagcggccgctgcaggcttcctcgctcactgactcgctgcgctcggtcgttcggctgcggcgagcggtatcagctcact
caaaggcggtaatacggttatccacagaatcaggggataacgcaggaaagaacatgtgagcaaaaggccagcaaaaggccaggaacc
gtaaaaaggccgcgttgctggcgtttttccacaggctccgcccccctgacgagcatcacaaaaatcgacgctcaagtcagaggtggcgaaac
ccgacaggactataaagataccaggcgtttc

Table S7: pSB1A3-J23106-B0034-mVenus-B0015

cccctggaagctccctcgtgcgctctcctgttccgaccctgccgcttaccggatacctgtccgcctttctcccttcgggaagcgtggcgctttctcata
gctcacgctgtaggtatctcagttcggtgtaggtcgttcgctccaagctgggctgtgtgcacgaaccccccgttcagcccgaccgctgcgccttat
ccggtaactatcgtcttgagtccaacccggtaagacacgacttatcgccactggcagcagccactggtaacaggattagcagagcgaggtatg
taggcggtgctacagagttcttgaagtggtggcctaactacggctacactagaagaacagtatttggtatctgcgctctgctgaagccagttacctt
cggaaaaagagttggtagctcttgatccggcaaacaaaccaccgctggtagcggtggtttttttgtttgcaagcagcagattacgcgcagaaaa
aaaggatctcaagaagatcctttgatcttttctacggggtctgacgctcagtggaacgaaaactcacgttaagggattttggtcatgagattatcaa
aaaggatcttcacctagatccttttaaattaaaaatgaagttttaaatcaatctaaagtatatatgagtaaacttggtctgacagttaccaatgcttaa
tcagtgaggcacctatctcagcgatctgtctatttcgttcatccatagttgcctgactccccgtcgtgtagataactacgatacgggagggcttacca
tctggccccagtgctgcaatgataccgcgagacccacgctcaccggctccagatttatcagcaataaaccagccagccggaagggccgagc
gcagaagtggtcctgcaactttatccgcctccatccagtctattaattgttgccgggaagctagagtaagtagttcgccagttaatagtttgcgcaa
cgttgttgccattgctacaggcatcgtggtgtcacgctcgtcgtttggtatggcttcattcagctccggttcccaacgatcaaggcgagttacatgatc
ccccatgttgtgcaaaaaagcggttagctccttcggtcctccgatcgttgtcagaagtaagttggccgcagtgttatcactcatggttatggcagca
ctgcataattctcttactgtcatgccatccgtaagatgcttttctgtgactggtgagtactcaaccaagtcattctgagaatagtgtatgcggcgaccg
agttgctcttgcccggcgtcaatacgggataataccgcgccacatagcagaactttaaaagtgctcatcattggaaaacgttcttcggggcgaa
aactctcaaggatcttaccgctgttgagatccagttcgatataacccactcgtgcacccaactgatcttcagcatcttttactttcaccagcgtttctgg
gtgagcaaaaacaggaaggcaaaatgccgcaaaaaagggaataagggcgacacggaaatgttgaatactcatactcttcctttttcaatatta
ttgaagcatttatcagggttattgtctcatgagcggatacatatttgaatgtatttagaaaaataaacaaataggggttccgcgcacatttccccgaa
aagtgccacctgacgtctaagaaaccattattatcatgacattaacctataaaaataggcgtatcacgaggcagaatttcagataaaaaaaatc
cttagctttcgctaaggatgatttctggaattcgcggccgcttctagagtttacggctagctcagtcctaggtatagtgctagctactagagaaagag
gagaaactcgagatgcaaaagagcaaaggcgaagaactgttcacgggtgtggttccgatcctggttgaactggatggcgatgtgaacggtca
taaatttagcgtgtctggtgaaggcgaaggtgatgcgacctacggcaaactgacgctgaaactgatttgcaccacgggtaaactgccggttccg

tggccgaccctggtgaccacgctgggttatggtctgatgtgtttcgcacgttacccggatcacatgaaacgccatgatttctttaaatctgcgatgcc
ggaaggctatgtgcaggaacgtaccatctttttcaaagatgatggtaactacaaaacccgcgcggaagttaaatttgaaggcgatacgctggtg
aaccgtattgaactgaaaggtatcgatttcaaagaagatggcaatattctgggtcacaaactggaatacaactacaacagtcataacgtgtaca
ttaccgccgataaacagaaaaacggtatcaaagcaaacttcaaaatccgtcacaacatcgaagatggcggtgttcagctggccgatcattac
cagcagaacaccccgattggcgatggtccggtgctgctgccggataatcattatctgagttaccagagcaaactgtctaaagatccgaatgaa
aaacgcgatcacatggttctgctggaatttgtgaccgcggccggcattacgcatggtatggatgaactgtataaataatgaggatccgctgtccg
ccaggcatcaaataaaacgaaaggctcagtcgaaagactgggcctttcgttttatctgttgtttgtcggtgaacgctctctactagagtcacactgg
ctcaccttcgggtgggcctttctgcgtttatatactagtagcggccgctgcaggcttcctcgctcactgactcgctgcgctcggtcgttcggctgcgg
cgagcggtatcagctcactcaaaggcggtaatacggttatccacagaatcaggggataacgcaggaaagaacatgtgagcaaaaggcca
gcaaaaggccaggaaccgtaaaaaggccgcgttgctggcgtttttccacaggctccgcccccctgacgagcatcacaaaaatcgacgctca
agtcagaggtggcgaaacccgacaggactataaagataccaggcgtttc

Table S8: pSB1A3-J23106-B0034-GFP-B0015

cccctggaagctccctcgtgcgctctcctgttccgaccctgccgcttaccggatacctgtccgcctttctcccttcgggaagcgtggcgctttctcata
gctcacgctgtaggtatctcagttcggtgtaggtcgttcgctccaagctgggctgtgtgcacgaaccccccgttcagcccgaccgctgcgccttat
ccggtaactatcgtcttgagtccaacccggtaagacacgacttatcgccactggcagcagccactggtaacaggattagcagagcgaggtatg
taggcggtgctacagagttcttgaagtggtggcctaactacggctacactagaagaacagtatttggtatctgcgctctgctgaagccagttacctt
cggaaaaagagttggtagctcttgatccggcaaacaaaccaccgctggtagcggtggtttttttgtttgcaagcagcagattacgcgcagaaaa
aaaggatctcaagaagatcctttgatcttttctacggggtctgacgctcagtggaacgaaaactcacgttaagggattttggtcatgagattatcaa
aaaggatcttcacctagatccttttaaattaaaaatgaagttttaaatcaatctaaagtatatatgagtaaacttggtctgacagttaccaatgcttaa
tcagtgaggcacctatctcagcgatctgtctatttcgttcatccatagttgcctgactccccgtcgtgtagataactacgatacgggagggcttacca
tctggccccagtgctgcaatgataccgcgagacccacgctcaccggctccagatttatcagcaataaaccagccagccggaagggccgagc
gcagaagtggtcctgcaactttatccgcctccatccagtctattaattgttgccgggaagctagagtaagtagttcgccagttaatagtttgcgcaa
cgttgttgccattgctacaggcatcgtggtgtcacgctcgtcgtttggtatggcttcattcagctccggttcccaacgatcaaggcgagttacatgatc
ccccatgttgtgcaaaaaagcggttagctccttcggtcctccgatcgttgtcagaagtaagttggccgcagtgttatcactcatggttatggcagca
ctgcataattctcttactgtcatgccatccgtaagatgcttttctgtgactggtgagtactcaaccaagtcattctgagaatagtgtatgcggcgaccg
agttgctcttgcccggcgtcaatacgggataataccgcgccacatagcagaactttaaaagtgctcatcattggaaaacgttcttcggggcgaa
aactctcaaggatcttaccgctgttgagatccagttcgatataacccactcgtgcacccaactgatcttcagcatcttttactttcaccagcgtttctgg
gtgagcaaaaacaggaaggcaaaatgccgcaaaaaagggaataagggcgacacggaaatgttgaatactcatactcttcctttttcaatatta
ttgaagcatttatcagggttattgtctcatgagcggatacatatttgaatgtatttagaaaaataaacaaataggggttccgcgcacatttccccgaa
aagtgccacctgacgtctaagaaaccattattatcatgacattaacctataaaaataggcgtatcacgaggcagaatttcagataaaaaaaatc
cttagctttcgctaaggatgatttctggaattcgcggccgcttctagagtttacggctagctcagtcctaggtatagtgctagctactagagaaagag
gagaaactcgagatgcgtaaaggagaagaacttttcactggagttgtcccaattcttgttgaattagatggtgatgttaatgggcacaaattttctgt
cagtggagagggtgaaggtgatgcaacatacggaaaacttacccttaaatttatttgcactactggaaaactacctgttccatggccaacacttgt
cactactttcggttatggtgttcaatgctttgcgagatacccagatcatatgaaacagcatgactttttcaagagtgccatgcccgaaggttatgtac
aggaaagaactatatttttcaaagatgacgggaactacaagacacgtgctgaagtcaagtttgaaggtgatacccttgttaatagaatcgagtta
aaaggtattgattttaaagaagatggaaacattcttggacacaaattggaatacaactataactcacacaatgtatacatcatggcagacaaac
aaaagaatggaatcaaagttaacttcaaaattagacacaacattgaagatggaagcgttcaactagcagaccattatcaacaaaatactcca
attggcgatggccctgtccttttaccagacaaccattacctgtccacacaatctgccctttcgaaagatcccaacgaaaagagagaccacatgg
tccttcttgagtttgtaacagctgctgggattacacatggcatggatgaactatacaaataataacggatccgctgtccgccaggcatcaaataaa
acgaaaggctcagtcgaaagactgggcctttcgttttatctgttgtttgtcggtgaacgctctctactagagtcacactggctcaccttcgggtgggc
ctttctgcgtttatatactagtagcggccgctgcaggcttcctcgctcactgactcgctgcgctcggtcgttcggctgcggcgagcggtatcagctca
ctcaaaggcggtaatacggttatccacagaatcaggggataacgcaggaaagaacatgtgagcaaaaggccagcaaaaggccaggaac
cgtaaaaaggccgcgttgctggcgtttttccacaggctccgcccccctgacgagcatcacaaaaatcgacgctcaagtcagaggtggcgaaa
cccgacaggactataaagataccaggcgtttc

Table S9: pSB1A3-J23106-B0034-mTurquoise-B0015

cccctggaagctccctcgtgcgctctcctgttccgaccctgccgcttaccggatacctgtccgcctttctcccttcgggaagcgtggcgctttctcata
gctcacgctgtaggtatctcagttcggtgtaggtcgttcgctccaagctgggctgtgtgcacgaaccccccgttcagcccgaccgctgcgccttat
ccggtaactatcgtcttgagtccaacccggtaagacacgacttatcgccactggcagcagccactggtaacaggattagcagagcgaggtatg
taggcggtgctacagagttcttgaagtggtggcctaactacggctacactagaagaacagtatttggtatctgcgctctgctgaagccagttacctt
cggaaaaagagttggtagctcttgatccggcaaacaaaccaccgctggtagcggtggtttttttgtttgcaagcagcagattacgcgcagaaaa
aaaggatctcaagaagatcctttgatcttttctacggggtctgacgctcagtggaacgaaaactcacgttaagggattttggtcatgagattatcaa
aaaggatcttcacctagatccttttaaattaaaaatgaagttttaaatcaatctaaagtatatatgagtaaacttggtctgacagttaccaatgcttaa
tcagtgaggcacctatctcagcgatctgtctatttcgttcatccatagttgcctgactccccgtcgtgtagataactacgatacgggagggcttacca
tctggccccagtgctgcaatgataccgcgagacccacgctcaccggctccagatttatcagcaataaaccagccagccggaagggccgagc
gcagaagtggtcctgcaactttatccgcctccatccagtctattaattgttgccgggaagctagagtaagtagttcgccagttaatagtttgcgcaa

cgttgttgccattgctacaggcatcgtggtgtcacgctcgtcgtttggtatggcttcattcagctccggttcccaacgatcaaggcgagttacatgatc
ccccatgttgtgcaaaaaagcggttagctccttcggtcctccgatcgttgtcagaagtaagttggccgcagtgttatcactcatggttatggcagca
ctgcataattctcttactgtcatgccatccgtaagatgcttttctgtgactggtgagtactcaaccaagtcattctgagaatagtgtatgcggcgaccg
agttgctcttgcccggcgtcaatacgggataataccgcgccacatagcagaactttaaaagtgctcatcattggaaaacgttcttcggggcgaa
aactctcaaggatcttaccgctgttgagatccagttcgatataacccactcgtgcacccaactgatcttcagcatcttttactttcaccagcgtttctgg
gtgagcaaaaacaggaaggcaaaatgccgcaaaaaagggaataagggcgacacggaaatgttgaatactcatactcttcctttttcaatatta
ttgaagcatttatcagggttattgtctcatgagcggatacatatttgaatgtatttagaaaaataaacaaataggggttccgcgcacatttccccgaa
aagtgccacctgacgtctaagaaaccattattatcatgacattaacctataaaaataggcgtatcacgaggcagaatttcagataaaaaaaatc
cttagctttcgctaaggatgatttctggaattcgcggccgcttctagagtttacggctagctcagtcctaggtatagtgctagctactagagaaagag
gagaaactcgagatggttagcaagggtgaagaactgttcaccggcgtcgtgccgattctggttgagctggatggtgatgtcaacggtcacaagt
ttagcgttagcggtgagggcgagggcgacgccacctacggtaaattgaccctgaagtttatctgcacgaccggtaagctgccggttccgtggcc
gaccctggtgacgactctgtcgtggggcgtgcaatgtttcgcgcgctatccggatcacatgaaacagcatgacttctttaagagcgcgatgccgg
aaggctacgttcaggaacgtacgatctttttcaaagacgacggtaactataagacccgcgcagaagtcaagttcgagggtgacacgctggtga
atcgtattgagctgaaaggtattgactttaaagaggacggtaacatcctgggtcacaaactggagtataattacttcagcgacaatgtgtacatca
ccgctgataaacagaaaaacggcattaaagcaaacttcaagatccgtcacaatattgaagatggcggcgtgcaattggccgatcactatcaa
cagaacaccccgattggcgatggtccggtcctgctgccagataatcactacttgagcacgcaatccaaactgtccaaagatccgaacgaaaa
acgtgaccacatggtcctgctggaatttgttaccgcggcgggtatcacgctgggtatggacgaactgtacaagtaattaacggatccgctgtccg
ccaggcatcaaataaaacgaaaggctcagtcgaaagactgggcctttcgttttatctgttgtttgtcggtgaacgctctctactagagtcacactgg
ctcaccttcgggtgggcctttctgcgtttatatactagtagcggccgctgcaggcttcctcgctcactgactcgctgcgctcggtcgttcggctgcgg
cgagcggtatcagctcactcaaaggcggtaatacggttatccacagaatcaggggataacgcaggaaagaacatgtgagcaaaaggcca
gcaaaaggccaggaaccgtaaaaaggccgcgttgctggcgtttttccacaggctccgcccccctgacgagcatcacaaaaatcgacgctca
agtcagaggtggcgaaacccgacaggactataaagataccaggcgtttc

Table S10: Proteins enriched in the commercial extract (derived from the comparison of the commercial extract and the shaking flask batches S5/L0.5)

Uniprot ID	Fold change	-log10(FDR)	Protein name	Gene name	Keyword
A0A140NFX5_ECOBD	2,715709578	2,715709578	**D-aminoacyl-tRNA deacylase**	**dtd** ECBD_4140, HO396_19315	amino-acid related
A0A140N9P3_ECOBD	2,254748493	2,254748493	**NADH-quinone oxidoreductase**	**nuoG** ECBD_1378, HO396_11215	ATP synthesis related
A0A140N3Q3_ECOBD	NaN	NaN	**L-lactate dehydrogenase**	**lldD** ECBD_0120, HO396_17700	carbohydrate metabolism and respiration
A0A140NA88_ECOBD	NaN	NaN	**Cytochrome bo(3) ubiquinol oxidase ...**	**cyoB** ECBD_3227, HO396_01970	carbohydrate metabolism and respiration
A0A140NAN1_ECOBD	1,535175058	1,535175058	**Quinone-dependent D-lactate dehydro...**	**dld** ECBD_1525, HO396_10490	carbohydrate metabolism and respiration
A0A140NCG6_ECOBD	2,400717199	2,400717199	Cytochrome bd ubiquinol oxidase sub...	**cydA** ECBD_2928, HO396_03500	carbohydrate metabolism and respiration
A0A140NCT2_ECOBD	2,127606292	2,127606292	**Fumarate reductase flavoprotein sub...**	**frdA** ECBD_3875, HO396_20675	carbohydrate metabolism and respiration
A0A140NDR8_ECOBD	2,715709578	2,715709578	**Ubiquinone/menaquinone biosynthesis...**	**ubiE** ECBD_4190, HO396_19055	carbohydrate metabolism and respiration
A0A140N599_ECOBD	1,769795266	1,769795266	**DNA topoisomerase 4 subunit B**	**parE** ECBD_0709, HO396_14780	DNA modification and replication
A0A140N626_ECOBD	2,372032264	2,372032264	**DNA gyrase subunit A**	**gyrA** ECBD_1429, HO396_10965	DNA modification and replication
A0A140N6C1_ECOBD	1,368085361	1,368085361	**DNA gyrase subunit B**	**gyrB** ECBD_0004, HO396_18280	DNA modification and replication
A0A140NB69_ECOBD	2,379969605	2,379969605	**Integration host factor subunit alp...**	**ihfA** himA, ECBD_1933, HO396_08640	DNA modification and replication
A0A140NBC7_ECOBD	1,436044176	1,436044176	**Chromosome partition protein MukE**	**mukE** ECBD_2672, HO396_04835	DNA modification and replication
A0A140NBR4_ECOBD	2,116329269	2,116329269	**Chromosome partition protein MukB**	**mukB** ECBD_2671, HO396_04840	DNA modification and replication
A0A140NCD3_ECOBD	1,92703912	1,92703912	**Recombination-associated protein Rd...**	**rdgC** ECBD_3268	DNA modification and replication
A0A140NCN5_ECOBD	1,524964845	1,524964845	**DNA polymerase III subunit gamma/ta...**	**dnaX** ECBD_3186, HO396_02170	DNA modification and replication
A0A140NCX5_ECOBD	1,522980698	1,522980698	**DNA topoisomerase 1**	**topA** ECBD_2348, HO396_06510	DNA modification and replication
A0A140NDN9_ECOBD	1,818400105	1,818400105	**DNA protection during starvation pr...**	**dps** pexB, ECBD_2811, HO396_04120	DNA modification and replication
A0A140NDT9_ECOBD	1,452719489	1,452719489	**Chromosome partition protein MukF**	**mukF** ECBD_2673, HO396_04830	DNA modification and replication
A0A140NDV2_ECOBD	2,545724335	2,545724335	**Integration host factor subunit bet...**	**ihfB** himD, ECBD_2683, HO396_04780	DNA modification and replication
A0A140NHJ4_ECOBD	2,036802479	2,036802479	**DNA helicase**	**uvrD** mutU, recL, ECBD_4228, HO396_18870	DNA modification and replication

A0A140N210_ECOBD	1,973292106	1,973292106	**SelB translation factor**	**selB** ECBD_0140, HO396_17605	other
A0A140N500_ECOBD	1,682729453	1,682729453	Multidrug efflux transporter EmrAB ...	**emrR** ECBD_1036, HO396_12955	other
A0A140N5K0_ECOBD	1,739020298	1,739020298	2-octaprenyl-6-methoxyphenyl hydrox...	**ubiH** visB, ECBD_0830, HO396_14020	other
A0A140N763_ECOBD	1,995921991	1,995921991	RNA binding S1 domain protein	ECBD_0338	other
A0A140N7J9_ECOBD	2,262035298	2,262035298	**Biotin carboxylase**	**accC** ECBD_0489, HO396_15875	other
A0A140N7L1_ECOBD	1,51110941	1,51110941	Amidohydrolase	**abgA** ECBD_2279, HO396_06865	other
A0A140N843_ECOBD	1,432249637	1,432249637	FAD-dependent 2-octaprenylphenol hy...	**ubiI** visC, ECBD_0831, HO396_14015	other
A0A140N8E8_ECOBD	2,127606292	2,127606292	(P)ppGpp synthetase I, SpoT/RelA	ECBD_0945, HO396_13430	other
A0A140N8K8_ECOBD	1,719258427	1,719258427	Sua5/YciO/YrdC/YwlC family protein	ECBD_2356, HO396_06465	other
A0A140N8W1_ECOBD	NaN	NaN	**Signal peptidase I**	**lepB** ECBD_1113, HO396_12540	other
A0A140N8Y0_ECOBD	2,372032264	2,372032264	**Fumarate hydratase class I**	**fumB** ECBD_2034, HO396_08130	other
A0A140N919_ECOBD	NaN	NaN	Ancillary SecYEG translocon subunit...	**yfgM** ECBD_1173, HO396_12250	other
A0A140N9K0_ECOBD	NaN	NaN	Amidohydrolase	**abgB** ECBD_2280, HO396_06860	other
A0A140N9T6_ECOBD	2,715709578	2,715709578	**Ubiquinone biosynthesis O-methyltra...**	**ubiG** ECBD_1428, HO396_10970	other
A0A140N9Z7_ECOBD	2,714499052	2,714499052	**NADH-quinone oxidoreductase subunit...**	**nuoF** ECBD_1377, HO396_11220	other
A0A140NA70_ECOBD	NaN	NaN	Cytochrome d ubiquinol oxidase subu...	**cydB** ECBD_2927, HO396_03505	other
A0A140NBI8_ECOBD	1,395578749	1,395578749	Mischarged aminoacyl-tRNA deacylase	**yeaK** ECBD_1857, HO396_09015	other
A0A140NBK6_ECOBD	NaN	NaN	**Protease 4**	**sppA** ECBD_1878, HO396_08910	other
A0A140NC78_ECOBD	NaN	NaN	PTS N-acetyl glucosamine transporte...	**nagE** ECBD_2983, HO396_03200	other
A0A140NCR0_ECOBD	1,826979921	1,826979921	**Ribonuclease R**	**rnr** ECBD_3855, HO396_20795	other
A0A140NDI9_ECOBD	1,575341538	1,575341538	**3-methyl-2-oxobutanoate hydroxymeth...**	**panB** ECBD_3485	other
A0A140NEB0_ECOBD	NaN	NaN	2-octaprenyl-3-methyl-6-methoxy-1,4...	**ubiF** ECBD_2989, HO396_03135	other
A0A140NEV1_ECOBD	2,036802479	2,036802479	**Probable cytosol aminopeptidase**	**pepA** ECBD_3776, HO396_21195	other
A0A140NF24_ECOBD	2,715709578	2,715709578	DNA-binding protein HU-alpha	**hupA** ECBD_4032, HO396_19880	other
A0A140NFA9_ECOBD	2,715709578	2,715709578	DNA-binding protein HU-beta	**hupB** ECBD_3215, HO396_02020	other
A0A140NFX6_ECOBD	NaN	NaN	**Membrane protein insertase YidC**	**yidC** ECBD_4327, HO396_18320	other
A0A140NHC7_ECOBD	1,399920367	1,399920367	GTP-binding protein TypA	**typA** ECBD_4156, HO396_19235	other
A0A140NHQ8_ECOBD	2,545724335	2,545724335	**ATP-dependent protease subunit HslV**	**hslV** ECBD_4092, HO396_19540	other
A0A140SS21_ECOBD	1,67112025	1,67112025	**23S rRNA (guanosine-2'-O-)-methyltr...**	**rlmB** ECBD_3854, HO396_20800	other
A0A140SSA6_ECOBD	1,975462238	1,975462238	**Xaa-Pro dipeptidase**	**pepQ** ECBD_4178, HO396_19115	other
TRMA_ECOBD	1,876494138	1,876494138	**tRNA/tmRNA (uracil-C(5))-methyltran...**	**trmA** ECBD_4059, ECD_03850, B21_03799	other
A0A140NEN6_ECOBD	1,54674934	1,54674934	**10 kDa chaperonin**	**groS** groES, ECBD_3889, HO396_20605	protein folding or unfolding
A0A140NF86_ECOBD	2,520931426	2,520931426	**ATP-dependent protease ATPase subun...**	**hslU** ECBD_4093, HO396_19535	protein folding or unfolding
A0A140N2S3_ECOBD	1,432249637	1,432249637	**50S ribosomal protein L22**	**rplV** ECBD_0436, HO396_16175	Ribosomal protein
A0A140N2T1_ECOBD	1,777900735	1,777900735	**50S ribosomal protein L6**	**rplF** ECBD_0446, HO396_16125	Ribosomal protein
A0A140N2Z9_ECOBD	1,739020298	1,739020298	**30S ribosomal protein S9**	**rpsI** ECBD_0517, HO396_15755	Ribosomal protein
A0A140N340_ECOBD	1,370183834	1,370183834	**50S ribosomal protein L27**	**rpmA** ECBD_0557, HO396_15555	Ribosomal protein
A0A140N3G7_ECOBD	1,706209039	1,706209039	**50S ribosomal protein L3**	**rplC** ECBD_0431, HO396_16200	Ribosomal protein
A0A140N3H4_ECOBD	1,721845921	1,721845921	**50S ribosomal protein L14**	**rplN** ECBD_0441, HO396_16150	Ribosomal protein
A0A140N3L9_ECOBD	1,871209857	1,871209857	**50S ribosomal protein L28**	**rpmB** ECBD_0089, HO396_17855	Ribosomal protein
A0A140N4K1_ECOBD	1,818400105	1,818400105	**30S ribosomal protein S3**	**rpsC** ECBD_0437, HO396_16170	Ribosomal protein
A0A140N4M0_ECOBD	2,511036592	2,511036592	**50S ribosomal protein L17**	**rplQ** ECBD_0457, HO396_16070	Ribosomal protein
A0A140N528_ECOBD	1,453504867	1,453504867	**30S ribosomal protein S19**	**rpsS** ECBD_0435, HO396_16180	Ribosomal protein

A0A140N537_ECOBD	1,874352239	1,874352239	**30S ribosomal protein S8**	**rpsH** ECBD_0445, HO396_16130	Ribosomal protein
A0A140N548_ECOBD	2,252064104	2,252064104	**30S ribosomal protein S4**	**rpsD** ECBD_0455, HO396_16080	Ribosomal protein
A0A140N5A3_ECOBD	2,09019138	2,09019138	**50S ribosomal protein L5**	**rplE** ECBD_0443, HO396_16140	Ribosomal protein
A0A140N5B4_ECOBD	2,116329269	2,116329269	**30S ribosomal protein S13**	**rpsM** ECBD_0453, HO396_16090	Ribosomal protein
A0A140N5D7_ECOBD	2,106162063	2,106162063	**50S ribosomal protein L21**	**rplU** ECBD_0556, HO396_15560	Ribosomal protein
A0A140N5K8_ECOBD	1,577896025	1,577896025	**50S ribosomal protein L4**	**rplD** ECBD_0432, HO396_16195	Ribosomal protein
A0A140N5L7_ECOBD	1,564050884	1,564050884	**50S ribosomal protein L24**	**rplX** ECBD_0442, HO396_16145	Ribosomal protein
A0A140N6T7_ECOBD	2,091658201	2,091658201	**50S ribosomal protein L19**	**rplS** ECBD_1080, HO396_12730	Ribosomal protein
A0A140N6W8_ECOBD	2,508907286	2,508907286	**30S ribosomal protein S7**	**rpsG** ECBD_0408, HO396_16305	Ribosomal protein
A0A140N6Y5_ECOBD	1,993382971	1,993382971	**30S ribosomal protein S10**	**rpsJ** ECBD_0430, HO396_16205	Ribosomal protein
A0A140N6Z2_ECOBD	2,114660618	2,114660618	**50S ribosomal protein L16**	**rplP** ECBD_0438, HO396_16165	Ribosomal protein
A0A140N6Z9_ECOBD	1,719258427	1,719258427	**30S ribosomal protein S5**	**rpsE** ECBD_0448, HO396_16115	Ribosomal protein
A0A140N711_ECOBD	2,033564102	2,033564102	**50S ribosomal protein L15**	**rplO** ECBD_0450, HO396_16105	Ribosomal protein
A0A140N7D8_ECOBD	2,127606292	2,127606292	**30S ribosomal protein S16**	**rpsP** ECBD_1077, HO396_12745	Ribosomal protein
A0A140N7G4_ECOBD	1,550848276	1,550848276	**50S ribosomal protein L30**	**rpmD** ECBD_0449, HO396_16110	Ribosomal protein
A0A140N7J1_ECOBD	1,673120024	1,673120024	**50S ribosomal protein L2**	**rplB** ECBD_0434, HO396_16185	Ribosomal protein
A0A140N7K8_ECOBD	1,714325265	1,714325265	**30S ribosomal protein S14**	**rpsN** ECBD_0444, HO396_16135	Ribosomal protein
A0A140N7L9_ECOBD	1,739020298	1,739020298	**30S ribosomal protein S11**	**rpsK** ECBD_0454, HO396_16085	Ribosomal protein
A0A140N811_ECOBD	1,555826867	1,555826867	**30S ribosomal protein S15**	**rpsO** ECBD_0575, HO396_15455	Ribosomal protein
A0A140N846_ECOBD	2,214717979	2,214717979	**50S ribosomal protein L25**	**rplY** ECBD_1472, HO396_10755	Ribosomal protein
A0A140N8B4_ECOBD	2,017062565	2,017062565	**30S ribosomal protein S21**	**rpsU** ECBD_0676, HO396_14955	Ribosomal protein
A0A140NBA5_ECOBD	1,818400105	1,818400105	**30S ribosomal protein S1**	**rpsA** ECBD_2684, HO396_04775	Ribosomal protein
A0A140NCE1_ECOBD	2,520931426	2,520931426	**30S ribosomal protein S22**	**sra** ECBD_2159, HO396_07470	Ribosomal protein
A0A140NDV1_ECOBD	2,201228736	2,201228736	**50S ribosomal protein L9**	**rplI** ECBD_3831, HO396_20915	Ribosomal protein
A0A140NF32_ECOBD	1,61938993	1,61938993	**50S ribosomal protein L11**	**rplK** ECBD_4050, HO396_19790	Ribosomal protein
A0A140NFK2_ECOBD	2,068401615	2,068401615	**30S ribosomal protein S2**	**rpsB** ECBD_3450, HO396_00845	Ribosomal protein
A0A140NFU3_ECOBD	2,092890112	2,092890112	**30S ribosomal protein S20**	**rpsT** ECBD_3593, HO396_00120	Ribosomal protein
A0A140NGG7_ECOBD	1,735318381	1,735318381	**30S ribosomal protein S6**	**rpsF** ECBD_3834, HO396_20900	Ribosomal protein
A0A140NGH1_ECOBD	1,975462238	1,975462238	**30S ribosomal protein S18**	**rpsR** ECBD_3832, HO396_20910	Ribosomal protein
A0A140NHV0_ECOBD	1,897705914	1,897705914	**50S ribosomal protein L34**	**rpmH** ECBD_4330, HO396_18305	Ribosomal protein
A0A140SS63_ECOBD	1,706209039	1,706209039	**50S ribosomal protein L7/L12**	**rplL** ECBD_4047, HO396_19805	Ribosomal protein
A0A140SS71_ECOBD	2,206783483	2,206783483	**50S ribosomal protein L31**	**rpmE** ECBD_4088, HO396_19560	Ribosomal protein
A0A140N4C9_ECOBD	2,167111388	2,167111388	**Ribosomal RNA small subunit methylt...**	ECBD_0794	Ribosome associated
A0A140N5Y7_ECOBD	1,311643666	1,311643666	**Ribosome-binding factor A**	**rbfA** ECBD_0573, HO396_15465	Ribosome associated
A0A140N989_ECOBD	1,844365916	1,844365916	**ATP-dependent RNA helicase SrmB**	**srmB** ECBD_1104, HO396_12585	Ribosome associated
A0A140NBQ1_ECOBD	2,038435631	2,038435631	**ATP-dependent RNA helicase RhlE**	**rhlE** ECBD_2826, HO396_04045	Ribosome associated
A0A140ND33_ECOBD	1,403819411	1,403819411	**Ribosomal RNA large subunit methylt...**	**rlmI** ECBD_2627	Ribosome associated
A0A140ND50_ECOBD	2,23285838	2,23285838	**Ribosomal RNA large subunit methylt...**	**rlmL** rlmKL, ECBD_2647, HO396_04960	Ribosome associated
A0A140NDB6_ECOBD	1,83519948	1,83519948	**50S ribosomal protein L10**	**rplJ** ECBD_4048, HO396_19800	Ribosome associated
A0A140NEN1_ECOBD	2,233174935	2,233174935	Ribosome-associated protein YbcJ	**ybcJ** ECBD_3130, HO396_02460	Ribosome associated
A0A140NHX7_ECOBD	1,849800897	1,849800897	**Der GTPase-activating protein YihI**	**yihI** ECBD_4162, HO396_19205	Ribosome associated
A0A140N2L7_ECOBD	1,804072352	1,804072352	**Ribonuclease PH**	**rph** ECBD_0083, HO396_17885	RNA modification and processing
A0A140N5F4_ECOBD	2,260642936	2,260642936	**Polyribonucleotide nucleotidyltrans...**	**pnp** ECBD_0576, HO396_15450	RNA modification and processing

A0A140N5P0_ECOBD	1,567846121	1,567846121	**Ribosomal RNA small subunit methylt...**	**rsmI** ECBD_0594, HO396_15355	RNA modification and processing
A0A140N6N0_ECOBD	2,254491907	2,254491907	**Phenylalanine--tRNA ligase alpha su...**	**pheS** ECBD_1931, HO396_08650	RNA modification and processing
A0A140N6U7_ECOBD	1,607545562	1,607545562	**Pseudouridine synthase**	**rluD** ECBD_1090, HO396_12675	RNA modification and processing
A0A140N719_ECOBD	1,903366827	1,903366827	**Ribosome maturation factor RimM**	**rimM** ECBD_1078, HO396_12740	RNA modification and processing
A0A140N7K6_ECOBD	2,486257014	2,486257014	**Ribonuclease G**	**rng** ECBD_0500, HO396_15835	RNA modification and processing
A0A140N7Y4_ECOBD	1,659661707	1,659661707	**Pseudouridine synthase**	**rsuA** ECBD_1474, HO396_10745	RNA modification and processing
A0A140N8N4_ECOBD	2,715709578	2,715709578	**Phenylalanine--tRNA ligase beta sub...**	**pheT** ECBD_1932, HO396_08645	RNA modification and processing
A0A140N998_ECOBD	2,068401615	2,068401615	**Ribonuclease 3**	**rnc** ECBD_1114, HO396_12535	RNA modification and processing
A0A140N9A7_ECOBD	1,416878204	1,416878204	**Translational regulator CsrA**	**csrA** ECBD_1029, HO396_13015	RNA modification and processing
A0A140N9H5_ECOBD	1,688346029	1,688346029	tRNA/rRNA methyltransferase	ECBD_1099, HO396_12610	RNA modification and processing
A0A140N9P4_ECOBD	2,553374934	2,553374934	**Dual-specificity RNA methyltransfer...**	**rlmN** trmG, ECBD_1169, HO396_12270	RNA modification and processing
A0A140N9R6_ECOBD	1,395578749	1,395578749	**Exoribonuclease 2**	**rnb** ECBD_2331, HO396_06595	RNA modification and processing
A0A140NAA1_ECOBD	2,447254088	2,447254088	**Ribonuclease E**	**rne** ECBD_2516, HO396_05625	RNA modification and processing
A0A140NB54_ECOBD	1,994193494	1,994193494	**RNA chaperone ProQ**	**proQ** ECBD_1809, HO396_09260	RNA modification and processing
A0A140NBF5_ECOBD	1,419769861	1,419769861	**Serine--tRNA ligase**	**serS** ECBD_2702, HO396_04685	RNA modification and processing
A0A140NCL7_ECOBD	2,715709578	2,715709578	**Pseudouridine synthase**	**rluB** ECBD_2353, HO396_06485	RNA modification and processing
A0A140NFF4_ECOBD	2,715709578	2,715709578	**Queuine tRNA-ribosyltransferase**	**tgt** ECBD_3255, HO396_01830	RNA modification and processing
A0A140NG24_ECOBD	1,659661707	1,659661707	**Poly(A) polymerase I**	**pcnB** ECBD_3476	RNA modification and processing
A0A140SSA5_ECOBD	1,977286864	1,977286864	**ATP-dependent RNA helicase RhlB**	**rhlB** ECBD_4260, HO396_18695	RNA modification and processing
A0A140N4Y7_ECOBD	2,235066468	2,235066468	**ATP-dependent RNA helicase DeaD**	**deaD** csdA, ECBD_0578, HO396_15435	SOS or stress response
A0A140NF66_ECOBD	2,097195799	2,097195799	**Catalase-peroxidase**	**katG** ECBD_4081, HO396_19595	SOS or stress response
A0A140NHF7_ECOBD	1,396921716	1,396921716	**LexA repressor**	**lexA** ECBD_3990, HO396_20100	SOS or stress response
A0A140N2U0_ECOBD	2,486257014	2,486257014	**DNA-directed RNA polymerase subunit...**	**rpoA** ECBD_0456, HO396_16075	transcription: regulation, initiation, termination
A0A140N3D6_ECOBD	1,566160693	1,566160693	**Catabolite activator protein**	**crp** ECBD_0391, HO396_16390	transcription: regulation, initiation, termination
A0A140N683_ECOBD	1,659661707	1,659661707	**RNA polymerase sigma factor RpoD**	**rpoD** ECBD_0674, HO396_14965	transcription: regulation, initiation, termination
A0A140N6I0_ECOBD	1,566006197	1,566006197	HTH-type transcriptional regulator ...	**galR** ECBD_0887, HO396_13725	transcription: regulation, initiation, termination
A0A140N6J0_ECOBD	2,068401615	2,068401615	**DNA-directed RNA polymerase subunit...**	**rpoZ** ECBD_0076, HO396_17915	transcription: regulation, initiation, termination
A0A140N6Q0_ECOBD	1,343326004	1,343326004	**Transcription elongation factor Gre...**	**greB** ECBD_0339	transcription: regulation, initiation, termination
A0A140N6R1_ECOBD	2,102483642	2,102483642	**DNA-binding protein**	**stpA** ECBD_1050, HO396_12880	transcription: regulation, initiation, termination
A0A140N749_ECOBD	NaN	NaN	**RNA polymerase sigma factor**	**rpoE** ECBD_1108, HO396_12565	transcription: regulation, initiation, termination
A0A140N785_ECOBD	1,688346029	1,688346029	**HTH-type transcriptional repressor ...**	**nanR** ECBD_0521, HO396_15735	transcription: regulation, initiation, termination
A0A140N7D2_ECOBD	NaN	NaN	Transcriptional regulator PhoB	**phoB** ECBD_0374, HO396_16475	transcription: regulation, initiation, termination
A0A140N7D6_ECOBD	2,537728387	2,537728387	**Transcription termination/antitermi...**	**nusA** ECBD_0571, HO396_15475	transcription: regulation, initiation, termination
A0A140N7P3_ECOBD	1,600500752	1,600500752	**RNA polymerase sigma-54 factor**	**rpoN** ECBD_0540, HO396_15640	transcription: regulation, initiation, termination
A0A140N937_ECOBD	1,413350845	1,413350845	DNA-binding transcriptional regulat...	**ydfH** ECBD_2099, HO396_07780	transcription: regulation, initiation, termination
A0A140NBI7_ECOBD	NaN	NaN	Transcriptional regulator, TetR fam...	ECBD_2027	transcription: regulation, initiation, termination
A0A140NCH9_ECOBD	2,260642936	2,260642936	**DNA-binding protein**	**hns** ECBD_2385, HO396_06315	transcription: regulation, initiation, termination
A0A140NCP4_ECOBD	2,417519113	2,417519113	**Transcription-repair-coupling facto...**	**mfd** ECBD_2487	transcription: regulation, initiation, termination
A0A140NF01_ECOBD	1,963684673	1,963684673	**Transcription termination factor Rh...**	**rho** ECBD_4257, HO396_18710	transcription: regulation, initiation, termination
A0A140NG87_ECOBD	NaN	NaN	**Transcriptional regulator MraZ**	**mraZ** ECBD_3536, HO396_00405	transcription: regulation, initiation, termination
A0A140NGM4_ECOBD	NaN	NaN	**HTH-type transcriptional regulator ...**	**ulaR** ECBD_3843, HO396_20855	transcription: regulation, initiation, termination
A0A140NH27_ECOBD	2,033564102	2,033564102	**DNA-directed RNA polymerase subunit...**	**rpoC** ECBD_4045, HO396_19815	transcription: regulation, initiation, termination
A0A140NHL8_ECOBD	2,09019138	2,09019138	**Transcription termination/antitermi...**	**nusG** ECBD_4051, HO396_19785	transcription: regulation, initiation, termination

A0A140NI00_ECOBD	1,671048798	1,671048798	**Transcription antitermination prote...**	**rfaH** ECBD_4183, HO396_19090	transcription: regulation, initiation, termination
A0A140SS80_ECOBD	2,476778848	2,476778848	**DNA-directed RNA polymerase subunit...**	**rpoB** ECBD_4046, HO396_19810	transcription: regulation, initiation, termination
A0A140N3T4_ECOBD	1,840087474	1,840087474	**Translation initiation factor IF-2**	**infB** ECBD_0572, HO396_15470	translation: regulation, initiation
A0A140N6C6_ECOBD	2,542539645	2,542539645	**Elongation factor 4**	**lepA** ECBD_1112, HO396_12545	translation: regulation, initiation
A0A140N6E7_ECOBD	2,010027151	2,010027151	**Peptide chain release factor 2**	**prfB** ECBD_0846, HO396_13935	translation: regulation, initiation
A0A140N7C8_ECOBD	1,714325265	1,714325265	**SsrA-binding protein**	**smpB** ECBD_1067, HO396_12795	translation: regulation, initiation
A0A140N9R4_ECOBD	1,827844667	1,827844667	**Translation initiation factor IF-3**	**infC** ECBD_1927, HO396_08670	translation: regulation, initiation
A0A140NCI6_ECOBD	1,352429122	1,352429122	**Elongation factor Tu**	**tuf** ECBD_4053, HO396_19775	translation: regulation, initiation
A0A140NCP8_ECOBD	1,500612867	1,500612867	**Ribosomal silencing factor RsfS**	**rsfS** rsfA, ECBD_3014, HO396_03015	translation: regulation, initiation
A0A140NFM7_ECOBD	2,553374934	2,553374934	**50S ribosomal protein L1**	**rplA** ECBD_4049, HO396_19795	translation: regulation, initiation
A0A140N775_ECOBD	1,32727143	1,32727143	**Malate dehydrogenase**	**mdh** ECBD_0511, HO396_15785	tricarboxylic acid cycle
A0A140N9G2_ECOBD	2,715709578	2,715709578	**Succinate--CoA ligase [ADP-forming]...**	**sucD** ECBD_2932, HO396_03475	tricarboxylic acid cycle
A0A140NA80_ECOBD	2,715709578	2,715709578	**Succinate dehydrogenase flavoprotei...**	**sdhA** ECBD_2937, HO396_03450	tricarboxylic acid cycle
A0A140NAN3_ECOBD	1,762171174	1,762171174	**Isocitrate dehydrogenase [NADP]**	**icd** ECBD_2463, HO396_05890	tricarboxylic acid cycle
A0A140NBF4_ECOBD	2,715709578	2,715709578	**Succinate--CoA ligase [ADP-forming]...**	**sucC** ECBD_2933, HO396_03470	tricarboxylic acid cycle
A0A140NC10_ECOBD	2,258292721	2,258292721	**Citrate synthase**	**gltA** ECBD_2941, HO396_03430	tricarboxylic acid cycle
A0A140NDX4_ECOBD	1,64536185	1,64536185	**Dihydrolipoyllysine-residue succiny...**	**odhB** ECBD_2934, HO396_03465	tricarboxylic acid cycle
A0A140NDZ9_ECOBD	2,233174935	2,233174935	**Succinate dehydrogenase iron-sulfur...**	**sdhB** ECBD_2936, HO396_03455	tricarboxylic acid cycle
A0A140NE66_ECOBD	1,781439302	1,781439302	**Oxoglutarate dehydrogenase (succiny...**	**sucA** ECBD_2935, HO396_03460	tricarboxylic acid cycle
A0A140NFP9_ECOBD	2,434120388	2,434120388	**Aconitate hydratase B**	**acnB** ECBD_3501, HO396_00580	tricarboxylic acid cycle
A0A140NGN0_ECOBD	1,948708918	1,948708918	**Aspartate ammonia-lyase**	**aspA** ECBD_3892, HO396_20590	tricarboxylic acid cycle
A0A140SS67_ECOBD	1,792683719	1,792683719	**Phosphoenolpyruvate carboxylase**	**ppc** ECBD_4068, HO396_19660	tricarboxylic acid cycle

Table S11: Proteins enriched in the shaking flask extracts S5/L0.5 (derived from the comparison of the commercial extract and the shaking flask batches S5/L0.5)

Uniprot ID	Fold change	-log10(FDR)	Protein name	Gene name	Keyword
A0A140N4Y5_ECOBD	1,629766172	1,629766172	**Aspartate-semialdehyde dehydrogenas...**	**asd** ECBD_0309	amino-acid related
A0A140N627_ECOBD	1,975462238	1,975462238	**S-adenosylmethionine synthase**	**metK** ECBD_0798, HO396_14190	amino-acid related
A0A140N770_ECOBD	2,503832349	2,503832349	**4-hydroxy-tetrahydrodipicolinate sy...**	**dapA** ECBD_1211, HO396_12065	amino-acid related
A0A140N7T1_ECOBD	1,651582735	1,651582735	**Succinyl-diaminopimelate desuccinyl...**	**dapE** ECBD_1218, HO396_12030	amino-acid related
A0A140NDW9_ECOBD	2,165393138	2,165393138	**2,3,4,5-tetrahydropyridine-2,6-dica...**	**dapD** ECBD_3453, HO396_00830	amino-acid related
A0A140NEB2_ECOBD	1,671048798	1,671048798	**4-hydroxy-tetrahydrodipicolinate re...**	**dapB** ECBD_3585, HO396_00160	amino-acid related
A0A140N783_ECOBD	2,505331739	2,505331739	**Glyceraldehyde-3-phosphate dehydrog...**	**gapA** ECBD_1865, HO396_08975	carbohydrate metabolism and respiration
A0A140NB59_ECOBD	2,004735299	2,004735299	**Phosphofructokinase**	**pfkB** ECBD_1922, HO396_08695	carbohydrate metabolism and respiration
A0A140N640_ECOBD	2,233174935	2,233174935	**2,3-bisphosphoglycerate-independent...**	**gpmI** gpmM, pgmI, ECBD_0113, HO396_17735	glycolytic process
A0A140N6G0_ECOBD	1,995921991	1,995921991	**Enolase**	**eno** ECBD_0950, HO396_13405	glycolytic process
A0A140N821_ECOBD	2,123646427	2,123646427	**Fructose-bisphosphate aldolase**	**fbaA** ECBD_0813, HO396_14115	glycolytic process
A0A140N8E1_ECOBD	2,672314268	2,672314268	**Phosphoglycerate kinase**	**pgk** ECBD_0812, HO396_14120	glycolytic process
A0A140N9C3_ECOBD	2,486257014	2,486257014	**Glucokinase**	**glk** ECBD_1284, HO396_11675	glycolytic process
A0A140N9D9_ECOBD	1,935051911	1,935051911	**2,3-bisphosphoglycerate-dependent p...**	**gpmA** ECBD_2912, HO396_03615	glycolytic process
A0A140N9V8_ECOBD	2,631339246	2,631339246	**Pyruvate kinase**	**pykF** ECBD_1969, HO396_08460	glycolytic process
A0A140NCD7_ECOBD	2,715709578	2,715709578	**Glucose-6-phosphate isomerase**	**pgi** ECBD_4012, HO396_20000	glycolytic process
A0A140NDL0_ECOBD	1,660343405	1,660343405	**Pyruvate dehydrogenase E1 component**	ECBD_3505	glycolytic process

A0A140NE27_ECOBD	1,831677521	1,831677521	**Acetyltransferase component of pyru...**	**aceF** ECBD_3504, HO396_00565	glycolytic process
A0A140NFX2_ECOBD	2,233174935	2,233174935	**Probable phosphoglycerate mutase Gp...**	**gpmB** ECBD_3625, HO396_21960	glycolytic process
A0A140N4P3_ECOBD	1,728448851	1,728448851	**4-hydroxy-3-methylbut-2-en-1-yl dip...**	**ispG** gcpE, ECBD_1171, HO396_12260	other
A0A140N5W3_ECOBD	1,91680254	1,91680254	Alanine transaminase AlaA	**alaA** ECBD_1369, HO396_11250	other
A0A140N6S0_ECOBD	1,799059234	1,799059234	**2-C-methyl-D-erythritol 2,4-cyclodi...**	**ispF** ECBD_0978, HO396_13270	other
A0A140N733_ECOBD	1,435570997	1,435570997	**2-C-methyl-D-erythritol 4-phosphate...**	**ispD** ECBD_0977, HO396_13275	other
A0A140N7Z8_ECOBD	1,812227448	1,812227448	**Dihydropteroate synthase**	**folP** ECBD_0565, HO396_15515	other
A0A140N8J7_ECOBD	1,343326004	1,343326004	**dTDP-4-dehydrorhamnose reductase**	**rfbD** ECBD_1615, HO396_10030	other
A0A140NAW9_ECOBD	1,65836961	1,65836961	**Adenylate kinase**	**adk** ECBD_3182, HO396_02190	other
A0A140NDD0_ECOBD	1,575341538	1,575341538	**Thymidylate kinase**	**tmk** ECBD_2503, HO396_05690	other
A0A140NDZ1_ECOBD	1,531912673	1,531912673	Thiamine pyrophosphate protein TPP ...	**poxB** ECBD_2723, HO396_04570	other
A0A140NFK0_ECOBD	2,258292721	2,258292721	**Methionine synthase**	**metH** ECBD_4018, HO396_19970	other
A0A140NGD5_ECOBD	1,663133441	1,663133441	**4-hydroxy-3-methylbut-2-enyl diphos...**	**ispH** lytB, ECBD_3587, HO396_00150	other
A0A140N457_ECOBD	2,434120388	2,434120388	**Guanylate kinase**	**gmk** ECBD_0077, HO396_17910	other

Table S12: Enriched proteins derived from the comparison of S0/L1 vs. S5/L1, S0/L1 vs. S15/L1 and of bioreactor S5/L1 against the commercial extract

S5/L1 vs. S0/L1

S5/L1 proteins in GO terms with GO FDR <0.05

Entry name	Protein name	Gene name	GO (biological process)	Keyword
S5/L1 proteins in GO terms with GO FDR < 0.05				
A0A140NAS5_ECOBD	Transcriptional regulator, AsnC fam...	ECBD_2706		Transcriptional regulator
A0A140N479_ECOBD	**DNA mismatch repair protein MutS**	**mutS** ECBD_0991	mismatch repair	DNA repair
A0A140NF57_ECOBD	Transcriptional regulator, LysR fam...	ECBD_4063		Transcriptional regulator
A0A140NFS3_ECOBD	Transcriptional regulator, LacI fam...	ECBD_4090	regulation of transcription, DNA templated	Transcriptional regulator
A0A140N8H2_ECOBD	**Nucleoid-associated protein YejK**	**yejK** ECBD_1471		DNA relaxation
A0A140N6R1_ECOBD	**DNA-binding protein**	ECBD_1050	regulation of transcription, DNA templated	Transcriptional regulator
A0A140NCD3_ECOBD	**Recombination-associated protein Rd...**	**rdgC** ECBD_3268	DNA recombination	DNA recombination

S0/L1 proteins in GO terms with GO FDR <0.05

none

S0/L1 vs. S15L1

S15/L1 proteins in GO terms with GO FDR <0.05

Entry name	Protein name	Gene name	GO (biological process)	Keyword
A0A140N231_ECOBD	Transcriptional regulator, IclR fam...	ECBD_0160	regulation of transcription, DNA-templated	Transcriptional regulator
A0A140N3Y5_ECOBD	Transcriptional regulator, LysR fam...	ECBD_0633		Transcriptional regulator

A0A140N479_ECOBD	**DNA mismatch repair protein MutS**	**mutS** ECBD_0991	mismatch repair	DNA repair
A0A140N6R1_ECOBD	**DNA-binding protein**	ECBD_1050	regulation of transcription, DNA-templated	Transcriptional regulator
A0A140N7H8_ECOBD	Transcriptional regulator, DeoR fam...	ECBD_0611		Transcriptional regulator
A0A140N7Q6_ECOBD	**DNA-binding protein Fis**	**fis** ECBD_0484		Transcriptional regulator
A0A140N7T4_ECOBD	**DNA topoisomerase 4 subunit A**	**parC** ECBD_0720	chromosome segregation; DNA topological change	DNA relaxation
A0A140N8H2_ECOBD	**Nucleoid-associated protein YejK**	**yejK** ECBD_1471		DNA relaxation
A0A140N8I2_ECOBD	Transcriptional regulator, DeoR fam...	ECBD_2335		Transcriptional regulator
A0A140N9L7_ECOBD	ROK family protein	ECBD_2986		Transcriptional regulator
A0A140NAS5_ECOBD	Transcriptional regulator, AsnC fam...	ECBD_2706		Transcriptional regulator
A0A140NB26_ECOBD	Transcriptional regulator, DeoR fam...	ECBD_1874		Transcriptional regulator
A0A140NBN2_ECOBD	CI repressor	ECBD_2773	negative regulation of transcription, DNA-templated; protein complex oligomerization	Transcriptional regulator
A0A140NBP1_ECOBD	**Sugar fermentation stimulation prot...**	**sfsA** ECBD_3473		Carbohydrate metabolism
A0A140NBR4_ECOBD	**Chromosome partition protein MukB**	**mukB** ECBD_2671	cell cycle; cell division; chromosome condensation; chromosome segregation; DNA replication	DNA replication
A0A140NC75_ECOBD	Phage shock protein B	ECBD_2312	phage shock; regulation of transcription, DNA-templated	Transcriptional regulator
A0A140NC83_ECOBD	**Transcriptional regulatory protein ...**	ECBD_2294	regulation of transcription, DNA-templated	Transcriptional regulator
A0A140NCD3_ECOBD	**Recombination-associated protein Rd...**	**rdgC** ECBD_3268	DNA recombination	DNA recombination
A0A140NDA9_ECOBD	Transcriptional regulator, LysR fam...	ECBD_3416		Transcriptional regulator
A0A140NDV2_ECOBD	**Integration host factor subunit bet...**	**ihfB** himD, ECBD_2683	DNA recombination; regulation of transcription, DNA-templated; regulation of translation	DNA recombination
A0A140NEJ9_ECOBD	Transcriptional regulator, IclR fam...	ECBD_3151	regulation of transcription, DNA-templated	Transcriptional regulator

S0/L1 proteins in GO terms with GO FDR <0.05

none

Bioreactor vs. Commercial extract

Bioreactor proteins with GO FDR <0.05

**Entry name**	**Protein name**	**Gene name**	**GO (biological process)**	**Keyword**
A0A140N487_ECOBD	**2-amino-3-ketobutyrate coenzyme A l...**	**kbl** ECBD_0108	biosynthetic process; L-threonine catabolic process to glycine	Amino acid related
A0A140N5K6_ECOBD	Endoribonuclease L-PSP	ECBD_0627		Nuclease
A0A140N6I5_ECOBD	**Selenide, water dikinase**	**selD** ECBD_1880	selenocysteine biosynthetic process	Other
A0A140N6K1_ECOBD	**Amino-acid acetyltransferase**	**argA** ECBD_0907	arginine biosynthetic process	Amino acid related
A0A140N725_ECOBD	**Phosphoadenosine phosphosulfate red...**	**cysH** ECBD_0967	hydrogen sulfide biosynthetic process; sulfate assimilation, phosphoadenylyl sulfate reduction by phosphoadenylyl-sulfate reductase (thioredoxin)	Other
A0A140N770_ECOBD	**4-hydroxy-tetrahydrodipicolinate sy...**	**dapA** ECBD_1211	diaminopimelate biosynthetic process; lysine biosynthetic process via diaminopimelate	Amino acid related

A0A140N7T9_ECOBD	Cystathionine beta-lyase	ECBD_0732	transsulfuration	Other
A0A140N8G9_ECOBD	**Sulfite reductase [NADPH] flavoprot...**	**cysJ** ECBD_0965	cysteine biosynthetic process; hydrogen sulfide biosynthetic process; sulfate assimilation	Amino acid related
A0A140N8V9_ECOBD	**Adenylyl-sulfate kinase**	**cysC** ECBD_0974	hydrogen sulfide biosynthetic process; sulfate assimilation	Other
A0A140NE69_ECOBD	**3-isopropylmalate dehydratase large...**	**leuC** ECBD_3544	branched-chain amino acid biosynthetic process; leucine biosynthetic process	Amino acid related
A0A140NEC9_ECOBD	**Homoserine O-succinyltransferase**	**metAS** ECBD_4024	L-methionine biosynthetic process from homoserine via O-succinyl-L-homoserine and cystathionine	Amino acid related

TXTL proteins with GO FDR <0.05

none

Table S13: Summary of protein numbers for each comparison: total number of proteins found in the extracts, number of unique proteins, number of proteins with FDR<0.05 and numbers of proteins in GO terms with GO FDR<0.05

Shaking flask vs. bioreactor

same lysis setting (S5/L1), different culture conditions

	SF S5/L0.5	BR S5/L0.5
total number of proteins	1480	1536
unique proteins	65	0
thereof n=3 n=0	11	0
Proteins with FDR <0.05	0	8
Proteins in GO with FDR <0.05	3	0
thereof n=3 n=0	3	0

S0/L1 vs S5/L1

same lysozyme concentration, but 0 vs 5 sonication cycles

	S0/L1	S5/L1
total number of proteins	1426	1469
unique proteins	42	85
thereof n=3 n=0	2	27
Proteins with FDR <0.05	0	0
Proteins in GO with FDR <0.05	0	7
thereof n=3 n=0	0	7

S0/L1vs S15/L1

same lysozyme concentration, but 0 vs. 15 sonication cycles

	S0/L1	S15/L1
total number of proteins	1426	1469

unique proteins	57	120
thereof n=3 n=0	3	47
Proteins with FDR <0.05	1	6
Proteins in GO with FDR <0.05	0	21
thereof n=3 n=0	0	19

commercial extract vs. Bioreactor S5/L0.5

	myTXTL	Bioreactor
total number of proteins	1566	1536
unique proteins	215	185
thereof n=3 n=0	121	30
Proteins with FDR <0.05	5	8
Proteins in GO with FDR <0.05	0	11
thereof n=3 n=0	0	5

commercial versus shaking flask S5/L0.5

	myTXTL	S5/L0.5
total number of proteins	1566	1480
unique proteins n=1,2,3 vs. n=0	214	128
thereof n=3 n=0	116	53
Proteins with FDR <0.05	309	356
Proteins in GO with FDR <0.05	172	31
thereof n=3 n=0	15	0

Supporting References

[1] Z. Z. Sun, C. A. Hayes, J. Shin, F. Caschera, R. M. Murray, V. Noireaux, *J. Vis. Exp.* **2013**, e50762.

[2] a) T. W. Kim, J. W. Keum, I. S. Oh, C. Y. Choi, C. G. Park, D. M. Kim, *J. Biotechnol.* **2006**, *126*, 554-561; b) Y. C. Kwon, M. C. Jewett, *Sci. Rep.* **2015**, *5*, 8663.

[3] M. K. Takahashi, J. Chappell, C. A. Hayes, Z. Z. Sun, J. Kim, V. Singhal, K. J. Spring, S. Al-Khabouri, C. P. Fall, V. Noireaux, R. M. Murray, J. B. Lucks, *ACS Synth. Biol.* **2015**, *4*, 503-515.

[4] M. Rustad, A. Eastlund, R. Marshall, P. Jardine, V. Noireaux, *J. Vis. Exp.* **2017**, 1 - 9.

[5] J. Garamella, R. Marshall, M. Rustad, V. Noireaux, *ACS Synth. Biol.* **2016**, *5*, 344-355.

[6] F. R. Blattner, B. G. Williams, A. E. Blechl, K. Denniston-Thompson, H. E. Faber, L. Furlong, D. J. Grunwald, D. O. Kiefer, D. D. Moore, J. W. Schumm, E. L. Sheldon, O. Smithies, *Science* **1977**, *196*, 161 - 169.

[7] a) J. r. Cox, N. Neuhauser, A. Michalski, R. A. Scheltema, J. V. Olsen, M. Mann, *J. Proteome Res.* **2011**, *10*, 1794-1805; b) S. Tyanova, T. Temu, J. Cox, *Nat. Protocols* **2016**, *11*, 2301-2319.

[8] J. Cox, M. Y. Hein, C. A. Luber, I. Paron, N. Nagaraj, M. Mann, *Mol Cell Proteomics* **2014**, *13*, 2513-2526.

[9] Y. Benjamini, Y. Hochberg, *J. Roy. Stat. Soc. B* **1995**, *57*, 289-300.

[10] D. W. Huang, B. T. Sherman, R. A. Lempicki, *Nat. Protocols* **2008**, *4*, 44-57.

[11] A. Bateman, M. J. Martin, S. Orchard, M. Magrane, R. Agivetova, S. Ahmad, E. Alpi, E. H. Bowler-Barnett, R. Britto, B. Bursteinas, H. Bye-A-Jee, R. Coetzee, A. Cukura, A. D. Silva, P. Denny, T. Dogan, T. G. Ebenezer, J. Fan, L. G. Castro, P. Garmiri, G. Georghiou, L. Gonzales, E. Hatton-Ellis, A. Hussein, A. Ignatchenko, G. Insana, R. Ishtiaq, P. Jokinen, V. Joshi, D. Jyothi, A. Lock, R. Lopez, A. Luciani, J. Luo, Y. Lussi, A. MacDougall, F. Madeira, M. Mahmoudy, M. Menchi, A. Mishra, K. Moulang, A. Nightingale, C. S. Oliveira, S. Pundir, G. Qi, S. Raj, D. Rice, M. R. Lopez, R. Saidi, J. Sampson, T. Sawford, E. Speretta, E. Turner, N. Tyagi, P. Vasudev, V. Volynkin, K. Warner, X. Watkins, R. Zaru, H. Zellner, A. Bridge, S. Poux, N. Redaschi, L. Aimo, G. Argoud-Puy, A. Auchincloss, K. Axelsen, P. Bansal, D. Baratin, M. C. Blatter, J. Bolleman, E. Boutet, L. Breuza, C. Casals-Casas, E. de Castro, K. C. Echioukh, E. Coudert, B. Cuche, M. Doche, D. Dornevil, A. Estreicher, M. L. Famiglietti, M. Feuermann, E. Gasteiger, S. Gehant, V. Gerritsen, A. Gos, N. Gruaz-Gumowski, U. Hinz, C. Hulo, N. Hyka-Nouspikel, F. Jungo, G. Keller, A. Kerhornou, V. Lara, P. Le Mercier, D. Lieberherr, T. Lombardot, X. Martin, P. Masson, *Nucleic Acids Res.* **2021**, *49*, D480-D489.

[12] P. Puigbo, I. G. Bravo, S. Garcia-Vallve, *Biol. Direct* **2008**, *3*, 38.

[13] A. E. Borujeni, A. S. Channarasappa, H. M. Salis, *Nucleic Acids Res.* **2014**, *42*, 2646-2659.

[14] D. S. Bindels, L. Haarbosch, L. van Weeren, M. Postma, K. E. Wiese, M. Mastop, S. Aumonier, G. Gotthard, A. Royant, M. A. Hink, T. W. Gadella, Jr., *Nat. Methods* **2017**, *14*, 53-56.

[15] G. J. Kremers, J. Goedhart, E. B. van Munster, T. W. Gadella, Jr., *Biochemistry* **2006**, *45*, 6570-6580.

[16] B. P. Cormack, R. H. Valdivia, S. Falkow, *Gene* **1996**, *173*, 33-38.

[17] J. Goedhart, D. von Stetten, M. Noirclerc-Savoye, M. Lelimousin, L. Joosen, M. A. Hink, L. van Weeren, T. W. Gadella, Jr., A. Royant, *Nat. Commun.* **2012**, *3*, 751.

[18] G. T. Hanson, T. B. McAnaney, E. S. Park, M. E. Rendell, D. K. Yarbrough, S. Chu, L. Xi, S. G. Boxer, M. H. Montrose, S. J. Remington, *Biochemistry* **2002**, *41*, 15477-15488.

[19] K. Fujiwara, N. Doi, *Plos One* **2016**, *11*, e0154614.
